# Spirit of the Foreign Periodicals, &c.

**Published:** 1837-04-01

**Authors:** 


### Spirit of the Foreign Periodicals, &c.

### Discovery of the Natural Cow-pox at Passy near Paris—Report of M. Bousquet.

The genuine cow-pox is of extremely rare occurrence among cattle. The cow is subject to other eruptions, besides the true one, on the udder; and these have very generally been mistaken for the genuine vaccinia. We have heard a great deal, of late years, of epidemics of the cow-pox in various parts of France and other countries; but with the exception of one or two, at most, of these alleged instances, we believe that the disease was in all of the spurious kind. We have visited many of the dairies, where it was reported that cow-pox prevailed; but hitherto we have not met with a single satisfactory case of the genuine eruption. Cows are very subject, during certain seasons, and especially after calving, to various eruptions on their teats—they are called by the country people "feux de lait." But these eruptions never exhibit those characters, which Jenner has very properly described as pathognomonic of the real and effective cow-pox. The vesicles of the latter always present a blueish or somewhat livid hue; they are surrounded with an erysipelatous redness, are apt to degenerate into troublesome phagedenic sores, and are usually accompanied with a good deal of disturbance of the animal's system.

It is probable that the genuine cow-pox is not nearly so rare as we suppose: possibly it is often overlooked; but most certain it is that, of late years, it has been seldom discovered in any dairy. Until last year, no authentic and quite trustworthy account of its having been observed in France, has been ever recorded; and in England, the parent country of cow-pox, the lymph, at present in use there, is that which has been kept up by successive vaccination of the human subject, from the time of Jenner. No doubt, if medical men paid more attention to the diseases of the lower animals, and were in the habit of visiting dairies for the purpose of discovering the genuine cow-pox, numerous opportunities of renewing the lymph from the original source might very possibly have been found: but so it is, as we have stated. The ancient committee of vaccination in France alluded to only one instance of the genuine cow-pox having been observed by any of their correspondents; and that instance is of very doubtful accuracy. It occurred in Prussia during the year 1812, and the account of it is far from being satisfactory. Since the date mentioned, a score, at least, of physicians in different parts of France have described instances of genuine cow-pox observed by them; but in all these, without exception, the failure of vaccination with the lymph has sufficiently proved the error of the announcement. In 1832, Dr. Maceroni of Rome examined several cows which had been recently sent from Switzerland, and which, it was said, were affected with cow-pox. Unfortunately the eruption had nearly passed away; but he obtained a few of the crusts of the pustules; these he beat down with water to the consistence of oil, and used the fluid, *successfully* in one or two cases, for the purposes of vaccination. We are not acquainted with the particulars of these experiments. The recently-published accounts of the genuine cow-pox in the beast at Wurtemburg, by Prof. Ering, and at Amiens by Dr. Autier, are far too meagre and imperfect to warrant reliance. I, says M. B., tried some of the lymph sent to Paris by Dr. Autier, but did not succeed with it in one operation.

In the beginning of 1836, the French journals teemed with reports, from different quarters, of the genuine cow-pox having been discovered. I visited a number of the places contiguous to Paris, and wrote to a great number of medical men residing in the more distant districts, where, it was alleged, that the disease existed; but not in one instance have I been satisfied of the existence of the genuine cow-pox. M. Dupuy accompanied me in several of the excursions I made round Paris, and he also was equally unconvinced.

We come now to mention the particulars of the recent *indubitable* discovery of the disease at Passy.* A countrywoman had gone to Chaillot, to consult Dr. Perdrau there. She was suffering from feverishness, and was otherwise indisposed. Dr. P. noticed some pustules on her hands; and on examining these, he was immediately struck with their resemblance to the genuine cowpox. He questioned her:—"Quel métier faites vous?—Je suis laitière. Avez vous remarqué au pis de vos vaches des pustules, à peu pres semblables à celles que vous me montrez?—Oui, Monsieur, et je crois bien que je les ai gagnées. Aviez vous des gercures aux mains?—Oui, Monsieur."

This account was sufficient to excite Dr. Perdrau's curiosity. He, therefore, sent the woman to be examined by M. Nauche, and by M. Bousquet (the author of the present memoir). They found three pustules on her right hand; one on the thumb, another on the back of the ring finger, and the third near the point of the fore-finger. There was also a similar pustule on the upper lip, near the right commissure of the mouth. The pustules were three or four lines in diameter, globular and prominent; their edges and the surrounding areolæ were of a blueish hue, such as I never observed before in cow-pox, but which at once recalled to my mind the description which Jenner has given of the pock on the cow. The usual appearances of the vesicles, as we now see them after vaccination—their central depression and their silvery white aspect—were very indistinctly marked; but this may have been partly owing to the advanced stage of their progress. The contents of the pustules were thick, yellowish-white, and purulent.

Nine children were inoculated with the matter, by three punctures on their left arms; and at the same time (but with another lancet), with ordinary vaccine lymph, by three punctures on their right arms. In one of these nine children, neither of the two inoculations produced any effect. In the other eight children, all the punctures on their right arms (with the ordinary lymph) succeeded, with the exception of one infant in whom one only of the punctures was followed by a vesicle. Of all the punctures on the left arm, (with the matter obtained from the pustules of the woman Fleury's hands,) three only, one in each of three children—Denis, Dubief, and Coussinet—had been followed by the formation of vesicles. These vesicles were in every respect similar to those on their right arms.

Four other children were inoculated on their left arms, with the matter taken from the vesicle on the child Denis' left arm, and in their right arms, with the matter from the vesicles on its right arm. Every one of both sets of punctures succeeded; those on the left, as well as those on the right, arms. Two children were inoculated in both arms, in presence of the vaccination committee, with the matter from the left arm vesicles only of Dubief and Coussinet; the wish of the committee being to discover the effects of the *new* lymph when inserted alone, and without the simultaneous insertion of the ordinary lymph.

Every one of these inoculations also succeeded perfectly.* Indeed the vesicles induced by the *new* lymph were altogether finer and more perfect—being larger, more depressed in their centres, of a more silvery hue, and more firm—than those induced by the *old* or ordinary lymph.

---

* This is a delightful village, situated upon an eminence on the banks of the Seine, about a league from Paris. There is a fine view of the Bois de Boulogne, and of the west end of Paris from it. It is noted, also, for its fine healthful air, and for some mineral springs, which have been long esteemed by invalids.

* The reader may be surprised at first, that so few of the punctures in the first series of cases—three only out of 24—succeeded, and that in the subsequent experiments every puncture should take.

This difference is quite what might have been expected, *a priori*. The matter obtained from the woman Fleury's hands was far advanced; it had become

The surrounding inflammation, too, was more severe and extensive, and the crusts were larger, thicker, and continued to adhere longer, than after ordinary vaccination. In some of the cases the areolar inflammation extended up the arm to the axilla, causing the glands there to become swollen and painful; and when the crusts fell off, the surface was found to be deeply ulcerated.

Jenner in his early experiments frequently met with the same occurrences; and hence he was often unwilling to make more than one puncture in each arm. M. Bousquet also found it necessary not to make more than two or three punctures with the *new* lymph; as the subsequent inflammation was sometimes apt to run too high.

So much for the external appearances of the vesicles produced by the *new* virus. It remained now to ascertain, whether the constitution of the children had been so affected by its operation, as to resist the influence of the variolous poison. There were two methods of ascertaining this; either by the direct inoculation of variolous matter into the arm, or by vaccinating with ordinary fresh vaccine lymph. The former test was no doubt the more decisive of the two; but there were difficulties to be overcome in using it. M. Bousquet therefore first tried the effects of re-vaccination on twelve children. In all of them the operation failed: there followed only a slight degree of inflammation around the punctures, but no vesicles were formed. M. B. then inoculated two children, who had been vaccinated with the *new* lymph, with small-pox matter; and still no effect was produced.

Since these experiments were made, M. B. has used the *new* lymph in several hundred cases, and with an almost uniform success. He has found, that he has much less often failed with the new lymph than with that in ordinary use.

He has also tried to re-vaccinate with the *new* lymph several persons who had, at former periods of their lives been vaccinated; and in a number of cases he has succeeded in producing perfect cow-pox vesicles. M. Boucher of Versailles re-vaccinated twelve persons, all of whom bore distinct marks of previous vaccination; and to render the experiment more instructive, he inserted the *new* lymph into the left arms, and the lymph in ordinary use in the right arms of his patients. The result was, that "le nouveau vaccine a produit de superbes pustules vaccinales sur toutes les personnes sans exception, et en nombre egal à celui des piqures; tandis que l'ancien vaccin n'a produit qu'une fausse vaccine, lorsqu'il n'est pas sur le champ et sans donner le moindre signe de reaction."

M. Bousquet, having thus indisputably proved the excellence of the *new* virus, proceeds to compare the vesicles produced by its inoculation with those described and figured in the early days of vaccination, and he finds a very remarkable resemblance between them. In both, the process of maturation has been slower, the surrounding inflammation more severe and diffused, and the falling off of the crusts more tardy, than in the ordinary vaccine vesicles seen now-a-days.

In conclusion, we may state that the cow, from which the woman Fleury was infected, was thin, and black, six or seven years old, and had calved six weeks before the appearance of any eruption on the udder. For two days, it would not eat any food, and the secretion of milk was greatly diminished. Several crusts were still on the udder, when M. Bousquet went to Passy. He requested that they might be preserved and sent to him, when they became detached; but his request was not attended to.—*Memoires de l'Academie Royale de Paris*, 1836.

---

nearly quite purulent; whereas the lymph employed in the second set of cases was taken on the seventh day after the vaccination. The older the lymph, the less active it is, the greater is the chance of failure with it, and the more necessary, therefore, it is to make numerous punctures. Two or three punctures are sufficient when we use early lymph, whereas ten or a dozen should be made, when the lymph is taken at a late date. Medical men are not sufficiently aware of this circumstance.

### Case of Glanders.

A middle aged robust man, who had been occupied with the care of some glandered horses in the Royal Veterinary College at Berlin, sickened on the 4th of October, but was not confined to his bed until the 9th. He was seized with frequently recurring fits of shivering, and with severe pains in the back and limbs. On the following day, he complained much of pain and sense of tightness in the chest, with difficulty of breathing and with cough, and these symptoms were accompanied with sharp febrile excitement. The thoracic distress became less; but as the fever did not abate, he was admitted into the hospital on the 18th.

He complained greatly of severe pains in the left leg (the inside of which was found to be erysipelatous), in the upper extremities, muscles of the back, and in the parietes of the chest. The disease resembled an attack of rheumatic fever: a warm bath, saline diaphoretics, and a blister to the chest were prescribed. During the night he was delirious: 12 leeches on the temples and cold lotion to the head were directed.

On the morning of the 21st, numerous boils, and dark red patches were observed on different parts of the surface. The largest one, three inches in length, was situated on the right cheek, and from the livid hue of the surface a tendency to gangrene was dreaded. The patient was oppressed and lethargic, the pulse was frequent, small and weak, and the skin was dripping with perspiration,

On the following two days, more furuncles made their appearance: the patient was alternately delirious and comatose. Some of the first crop of boils indicated the formation of matter. Mineral acids and wine were freely administered; but all the worst symptoms of typhus gravior, such as subsultus tendinum, convulsions, picking at the bed-clothes, &c. set in, and the man died on the evening of the 23rd.

*Dissection.* Some of the boils were found to contain pus; and in the intervals between them, in some parts, were observed numerous solitary pustules, not unlike those produced by vaccination. An incision into the inflamed spot on the left leg discovered an abscess, which contained about five ounces of consistent yellow pus. The apices of both lungs were softened. On the inner surface of the right lobe, there was an abscess. The head was not examined.—*Medicinish. Jahrbucher.*

We are surprised that the report of the appearances on dissection is so miserably scanty and imperfect. Not only should the viscera have been carefully examined, but the blood and other fluids ought to have been diligently observed and analyzed in such a case. There cannot be a reasonable doubt, that the preceding was an example of genuine, we do not say, exclusive, *humoral* disease.—*Rev.*

---

### Example of Spontaneous Combustion. Remarks upon this extraordinary Occurrence.

An old man 73, and his wife 65, years of age, had both been long immoderately addicted to the use of spirituous drinks. On the 6th of the present month (Sept. 1836) they had indulged in most excessive intoxication; and on the following morning, they were found dead.

M. Delaville, the "Procureur du Roi," invited M. Soly, the narrator of the case, to accompany him to the examination of the bodies.

The house was situated in the Commune de Surville, and about a quarter of a league from the Pont L'Eveque. On entering the room, some pieces of furniture were observed to be thinly covered with a grey coloured soot: it was most apparent on the windows, and on the glasses in the room. There was a strong empyrumatic smell; and on the floor, between a table on which there were some bottles and glasses containing brandy, and the ashes in the fire-place, lay a shapeless carbonized mass, in which four human legs could be recognised.

Two of the limbs were covered with black woollen stockings, and with cloth boots. One of the stockings was partially burnt at its upper part. The portions of the skin, protected by the boots, were reddened; the subjacent tissues did not exhibit any unusual appearances. Three inches or so above the knees, the thighs were converted into a black, carbonized, amorphous mass. There were no discoverable traces of the organs of generation, or of the pelvis (with the exception of the superior edge of the left os ilium), or of its contents; save only of the left ovary, which was found enveloped within an oleaginous and fetid charcoaly substance. At the level of the lower lumbar vertebræ, there was a solution of continuity between the upper and lower halves of the body; and the appearance of the parts indicated that this corpse had been laid across the other one below, and that thus the weight of the head and trunk had caused it to fall forwards, and to crack, in two, at the lumbar vertebræ. The two or three vertebræ which had become in consequence exposed to the air, were completely calcined.

The whiteness of these bones contrasted singularly with the dark pitchy mass, into which the thorax and its contents had been converted. The sternum and the greater number of the ribs had been quite destroyed; the blackened vertebral extremities of the first two or three left ribs still adhered to the spine. The cervical vertebræ were calcined, and terminated superiorly in a white, rounded and incinerated mass, which proved to be the cranium. This osseous case was so friable, that, in the attempt to lift it up, it crumbled to pieces; the lower jaw alone, which rested on the floor, retained its form.

Under the remains of this corpse, lay, at a right angle with it, the debris of a second. The left lower extremity was bare, spotted over on its front with vesicles, which contained a reddish coloured serum; posteriorly, along its whole extent, burned and scorched to the very bone. It was almost quite detached from the pelvis, and exsuded at the upper part a most fetid oleaginous fluid. The right limb, which, with the knee directed upwards, rested on the ground by the sole of its foot, was nearly in the same condition. On its anterior surface, it was covered with numerous phlyctenæ, some of which were observed on the sole of the foot. The ankle-joint was infiltrated with a dark serosity. The knee-joint was laid open, was of a yellow colour, and quite desiccated. The femur had snapped across two or three inches above the joint. From about this point in both limbs, the thighs were converted into a confused mass of unctuous charcoal. The pelvis and its contents had quite disappeared. Immediately above this point, the two corpses lay, the one across the other. It was not easy to separate the adhesions between them, formed by the slow and stifled combination of the bodies, and of their clothes, at this part. The right lung, and the liver of the under body had been so well protected by the carbonized layers of the clothes, and by the superimposed mass of the other corpse, that they could be readily recognized. They had shrunk to about one half of their usual size; their surfaces were hard, shining, and brittle. When cut across, they exhibited the consistence of firm cheese. The vertebral column and the ribs were reduced to a charcoal of a denser consistence, than that resulting from the combustion of the softer parts. The head was entire, but so besmeared with sooty matter, and so much disfigured, that the features could not be recognized. The projection of the nose, and the hollows of the orbits were sufficiently distinct; but, on the slightest shock, the bones gave way and broke down into numerous pieces. The brain, shrivelled and as hard as a piece of horn, was not larger than a hen's egg; it rested on the foramen magnum of the occipital bone. Of the upper extremities of both bodies, the only remains were a calcined portion, of about three or four inches in length, of one os humeri, and a few of the metacarpal bones: and these were quite incinerated. The whole weight of the debris of the two bodies, (omitting the lower extremities which were much less destroyed than other parts,) did not exceed four pounds. The period of time, during which the combustion had taken place, could not have exceeded twelve or thirteen hours.

The debris, stretched out on the floor which was soiled with a stinking, greasy fluid, were " encadrés" by the following objects. There was a table close to the feet, which were the parts furthest removed from the fire; this had escaped uninjured. Near to the two heads was the fire-place, and also a garde-cendre, and a gridiron, which had fallen down under the body of the woman: between the two heads there was a brand of wood still ignited (un tison encore enflammé). On the right side of the bodies, the only object was a single shoe; and on the left, between the lower extremities of the man and the wall, was a chair, which was but very partially scorched.—*Journal des Connois. Medico-Chirurg. Sept.* 1836.

Most of our readers are probably aware, that the recorded cases of spontaneous combustion of human beings have been considered by many intelligent men to be apocryphal; and in truth we cannot be much surprised at this opinion, when we reflect upon the extreme difficulty of burning to ashes recently dead animal matter. But such incredulity in the present day can be founded only on ignorance. The examples which have occurred of late years are too well authenticated to warrant the smallest doubt of the perfect accuracy of the descriptions given.

M. M. Dupuytren and Marc have detailed the history of two cases, which they themselves had an opportunity of examining. Dr. Apjohn of Dublin has published the reports of four cases, which have occurred recently in different parts of Ireland; and there was a case communicated three or four years ago to our cotemporary "The Medical and Surgical Journal," by a Mr. Devonald of London.

There cannot, therefore, be any rational doubts as to the truth of the fact—that human bodies have, in some manner, caught fire, and have partially or almost completely consumed away, leaving no remains but a mass of incinerated grease.

All the subjects of this horrid death have been notorious drunkards; and it is worthy of notice that most of them have been old corpulent women, addicted to intoxication.*

In no case, has the entire body been destroyed. Some portion or portions have remained only scorched; while the rest has been reduced to a heap of ashes, mixed with a black unctuous mass. The parts, which are usually not consumed, are the extremities, especially the hands and feet, and portions of the spine, and of the cranium. It is rare that the furniture of the room, even that in contact with the body, is much damaged. Sometimes, indeed, the bed, chair, &c. near the body, is scorched, but it is seldom or never consumed. In general the clothes, however, of the victim are found destroyed. A thick, greasy, and offensive soot usually covers all the objects around the corpse. In a few cases, the animal body has been found in the very act of combustion. A flickering, blue-coloured flame has been seen to be emitted from it; and we are assured by some writers, that water sprinkled upon the flame has seemed to enliven rather than to extinguish it.

In the majority of the cases on record, the unhappy victim has been seated near to a flame or fire, when the combustion had commenced. It seems therefore probable, that the ignition, so to speak, has not taken place spontaneously, but had been caused by the approach of the person to the fire or candle.

MM. Cat, Kopp, and Marc, however, are of opinion, from the history of certain cases, that the ignition may commence primarily in the animal body itself, without the application or approach of any heated, or burning body. It is well know that many dry vegetable matters, and that some minerals also will of themselves, under certain circumstances, become heated and catch fire. Some chemical mixtures too, we are aware, will occasion an immediate ignition. Thus, when together sulphuric and nitric acids are added to some essential oils, the whole becomes instantaneously ignited. It is upon these, and similar facts, that the writers last named rest in part their belief, that animal bodies may spontaneously ignite.

The development of the electrical spark on the one hand, and, on the other hand, the imbibition of all tissues

* Of 25 cases collected by Kopp and Lair, 24 have occurred in females.

of the body with spirituous liquid, especially when, at the same time, there is a quantity of fatty matter in the cellular substance, are the circumstances which some physicians have considered to be sufficient to occasion, and to keep up, the combustion of the human frame.

That alcoholic liquors may be very quickly absorbed into the blood, and thus be diffused through every part of the body, is known to all experienced pathologists. For example: a man took a wager that he could drink an enormous quanty of wine and brandy within a certain time. He did the feat, but killed himself by it. MM. Cuvier and Dumeril examined the body. They were struck with the strong alcoholic exhalation from every part. Again, M. Breschet mentions that he has repeatedly observed the same phenomenon in the bodies of criminals, who have been executed; and he adds this important remark, that fat animal substances are always found to burn away much more quickly and completely, than those which are lean. He has noticed this fact very frequently in the Anatomical Theatres of Paris, when he has wished to get rid of any portions of the bodies in the dissecting-rooms.

M. Marc concludes, from numerous observations, that an animal body may engender and become impregnated with inflammable gaseous matter; and that this may ignite, either on the approach of any lighted body, or by the action of an internal electrical current.

A case recently occurred in the Hôtel Dieu, which may be adduced, as affording some degree of probability to M. Marc's opinion. A man died suddenly in the hospital. The body was not examined for eight days after death: it was much decayed and putrid. The whole of the surface was ephysematous and covered with vesicles, some containing a bloody fluid, others filled with gas. The cavities of the abdomen, thorax, and also the ventricles of the brain, when cut into, emitted a quantity of gas; which, on the approach of a lighted candle, caught fire, and burned with a blueish flame. The gaseous matter is in all probability carburetted hydrogen. It is not improbable that the still more inflammable gas, the phosphuretted hydrogen, may be generated in the animal system. We know that this gas ignites on simple contact with the atmospheric air.

In whatever manner we endeavour to explain the occurrence of spontaneous combustion, there are one or two circumstances which strongly distinguish it from ordinary combustion.—Thus the rapidity, with which the decomposition and destruction of the body occurs, is very remarkable—a few hours are sufficient for this purpose. Again, how are we to account for the flame, which has been described as seen issuing from the body, not being communicated to inflammable substances placed in its vicinity?

As illustrative of many of the preceding remarks, we shall give the particulars of one of the cases narrated by Dr. Apjohn.

"A woman of about sixty years of age, who lived with her brother in the County of Down, retired one evening to bed with her daughter; both being, as was their constant habit, in a state of intoxication. A little before day, some members of the family were awakened by an extremely offensive smoke, which pervaded their apartment; and on going into the chamber, where the old woman and her daughter slept, they found the smoke to proceed from the body of the former, which appeared to be burning with an internal fire. It was as black as coal, and the smoke issued from every part of it. The combustion having been arrested, which was effected with difficulty, although there was no flame, life was found completely extinct. While the body was being removed into the coffin, which was done as soon as possible, it was dropping in pieces. Her daughter, who slept in the same bed, sustained no injury; nor did the combustion extend to the bed, or bed-clothes, which exhibited no other traces of fire, than the stains produced by the smoke.—According to the testimony of one of the relatives, there was no fire whatever in the room.

The subject of this case had been grossly intemperate for several days before her decease."

### Signor Catanoso's Case of Ligature of the Subclavian Artery, below the Clavicle.

A middle-aged man was admitted in Sept. 1835 into the hospital at Messina, with an extensive and deep cut in the axilla, which he had received three days before. He had fallen from a tree, and a sharp stump of a branch had penetrated deep into the armpit. The wound bled copiously at first, but had ceased spontaneously. The hæmorrhage had recurred several times. It was not suspected on the patient's admission, that the axillary artery had been wounded.* The compresses, however, which had been applied over the wound, were continually wet with the oozing blood; and when some clots were removed, there was exposed a tolerably deep excavation, which had already begun to suppurate. On the 14th day after the accident, an alarming hæmorrhage took place, and Signor Catanoso determined to put a ligature round the subclavian artery below the clavicle. A semicircular incision, pointing downwards, was made through the integuments, and the pectoral muscle having been then divided along the same line, the operator arrived at a tough inextricable fibro-cellular tissue, loaded with fat. This part of the dissection required great care, and occupied a long time. At length, the superior edge of the pectoralis minor muscle was reached, and the pulsations of the artery were felt. It was surrounded with its large accompanying vein, and the axillary plexus of nerves. These were at length separated from it with no small difficulty, and the aneurism needle, provided with one small but strong thread, was passed round it. When the ligature was tied, its two ends were cut off. The wound was then dressed simply, and the arm confined to the side. The pulse in the brachial artery at this time was not to be felt. At first, the wound appeared to be disposed to heal by the first intention; but in the course of two or three days it began to suppurate, and discharged a quantity of sanious offensive pus, mingled with some coagula of blood. The wound required to be enlarged to give a free issue to the discharge, and compresses and bandages were applied over every part, where the pus was disposed to collect. By the diligent use of these means, the state of the patient improved considerably, until the 19th day after the operation, when an arterial hæmorrhage took place. The clots being removed, the spot, whence the blood proceeded, was filled with the styptic powder of Bonafoux, (composed of colophony, charcoal, and gum arabic,) and compresses of lint were then secured over it, by means of a bandage applied tightly round the shoulder.—Fortunately the hæmorrhage did not return, and the wound slowly healed up. The cicatrisation was not however complete for nearly four months.

Signor Catanoso has appended to the report of the preceding case a long and very elaborate memoir on the ligature of wounded arteries, and on the best method of securing the subclavian. We are much pleased to find that his views on all practical points entirely coincide with the opinions and practice of the best English surgeons. He seems to be well acquainted with some of the works of our most esteemed authors, as Hodgson, Cooper, Jones, and others. The operation of tying the artery below the clavicle is seldom attempted in the present day; the extreme difficulty of reaching the artery, and the circumstance of its being quite enveloped with large nerves and veins, are powerful objections to this method. It is much easier to reach the artery above the clavicle. Whether the secondary hæmorrhage in the preceding case came from the tied trunk, or from some smaller vessel, is uncertain. We think the latter supposition the more probable, seeing that the bleeding ceased by the use of the styptic powder and of compresses.

The pamphlet, from which we have drawn the preceding short account, was published last year at Messina. We return our best thanks to Signor Catanoso for having transmitted us a copy, and invite him to send us occasionally such Italian works, as he may wish to be made known in this country.

* It is not stated in the report what was the state of the pulse at the wrist at this time.

### Cholera at Naples.

Dr. Zarlenga of Naples, Editor of the Italian Medical Journal called "Il Severino," has addressed to his distinguished countryman Assalini a letter, in which he has developed his views respecting the nature or proximate cause of the cholera—that singular scourge, which after having visited all the Northern countries of Europe three years ago, and then crossing the Atlantic Ocean, and inflicting its pestilence on the shores of America, has within the last twelve months wheeled back in its career, and asserted its dominion over the fair lands of Italy and Spain.

Dr. Zarlenga has politely sent his letter to our care, to be presented to the Medico-Chirurgical Society of London.

At the date of its subscription, September 1836, the author had not had any opportunities of witnessing* the disease, but he had carefully studied the descriptions given of it by the various authors of different countries, and he has drawn his conclusions from an impartial and minute examination of these.

He takes a rapid review of its first appearance, as a wide-spread epidemic, in Bengal in the year 1817, of its gradual diffusion over Hindostan, Persia, and other countries of Asia, and of its subsequent march Westward over Europe.

After noticing the symptoms of the disease, and the morbid appearances found on dissection in the bodies of those who have fallen victims to it, he proposes the interesting question,—what is the primary cause of the malady?

Dr. Zarlenga professes himself to be a disciple of the humoral pathology on this occasion. He believes that the disease is produced by the introduction from without into the circulation of some poisonous agent, which changes and corrupts the state of the blood; and with considerable ingenuity our author explains by this doctrine the very singular phenomena which so strikingly distinguish cholera from every other pestilence.

It is but fair to give the author's opinions on this subject in his own words.

"Talibus cadavericis observationibus innixus, hujusmodi morbi pathologicam conditionem in sanguine insitam fuisse censeo, cujus crasis per aerem inspiratum peculiarem conditionem nimis labefactam, maximeque turbatam hunc fluidum minus aptum ad vitam reddit; ita ut, super nervos ac fibras morbosam actionem dein explicans, talia causantur symptomata quæ nuper descripsimus, choleramque efficit. Hac crasi corrupta fit, ut sanguinis serosa pars e cruore disjungatur, ac ad tubum gastricum congregetur; ex quo evenit, ut materia serosa continuo vomatur aut per alvum ejiciatur, dummodo membranæ serosæ, atque vesica urinaria liquore omnino carens exarescit." By the aid of this hypothesis Dr. Zarlenga is of opinion that he can give a rational explanation of the leading pathological changes which are found during life and after death in cases of cholera—as, for example, the tarry uncoagulable state of the blood, the gorged state of the viscera, the blueish discoloration and livid spots on the skin, &c. &c. He traces an analogy between this new scourge of the nineteenth century and various other epidemic or endemic pestilences which have been long known, as the plague and yellow fever; and he suggests that the cause of each and all of them is to be sought for in certain morbific changes of the blood, induced by the absorption of some atmospheric poison into the system.

As to the alleged contagiousness of cholera, Dr. Zarlenga confesses that, from all that he has read, he is not inclined to believe in it.

With respect to the treatment suggested by Dr. Zarlenga, it is unnecessary for us to allude to it, as he had

* Since that period the cholera has not spared Naples and its vicinity. By the public prints we observe that it has caused great alarm, and not a little devastation, among the laughter-loving people of that gayest of cities.

not had an opportunity of testing its efficiency in actual practice.

No doubt, ere this time, Dr. Z. has seen the disease which he has so clearly described, and has tried those remedies, which he was induced, by reasoning on its probable causes, to recommend.

It will always give us the greatest pleasure to be the means of introducing Dr. Zarlenga to the attention of the British medical public, and we therefore invite him, and all our professional brethren in Italy, to forward any recent publications to our care.

---

ABSTRACT OF M. CHOMEL'S CLINIQUE AT THE HÔTEL DIEU.—FEVERS, CONTINUED AND INTERMITTENT—EXANTHEMATA — PLEURITIS AND PNEUMONIA — FATAL CASES OF ACUTE RHEUMATISM, AND POST-MORTEM APPEARANCES — CONNEXION OF CEREBRAL WITH PULMONIC DISEASE.

The Report embraces the period between November 1st, 1835, to August 1st, 1836. Four hundred and one patients were admitted during this time.

Of typhoid fever, there were 47 cases; and of these 13 proved fatal. Thirty-two of the patients were males, and 15 females. In 45, the age varied from 15 to 35; and in the remaining two, it was above 40. In general it was remarked, that the older the patient was, the more severe were the symptoms. The same observation has been made in preceding years. As respects the season of the year, 26 of the cases were admitted during the Winter, and 21 during the Summer months.

Not one of the 47 patients was a native of Paris: 26 of them had not been a whole year resident there. The mortality is in general greater among the new comers, than among the old residents.

In only one case, was there good ground for suspecting the origin of the fever in contagion. In 12 cases, the fever assumed a decidedly ataxic character: eight of these proved fatal.

In the remaining 35 cases, the adynamic form predominated; but with varying intensity in different patients.

As regards the diagnosis, the pathognomic characters were distinct in 40, at least, of the cases. In all these,* there was an intense febrile action, prostration of strength, headache, stupor and delirium; the tongue was dry, or gluey; the thirst great; the abdomen distended (meteorisé) and gurgling on pressure; and the bowels more or less relaxed. In most of the cases, there were red discoloured patches observable on some part of the surface, and a sibilant rale was heard on auscultating the chest. Whenever this assemblage of symptoms was present in a person, who was not affected with any phlegmasia of the head, chest, or venous system, there could be no doubt as to the nature of the case.

Pneumonia, indeed, and peritonitis, and also phlebitis, do occasionally appear under an ataxic form; but in all such cases the "point de depart" may generally be discovered: not so with genuine typhus.

In the seven other cases, the diagnosis was obscure. Two of these proved fatal; and in one of them, the obscurity was not removed by dissection; no lesion whatever being discovered in any part. In every other fatal case, there was found some lesion of the intestinal mucous surface, and generally of the glandulæ Peyeri.

A tendency to hæmorrhage from some part, as from the nose, bowels, and the subdermoid tissue, was present in several of the patients.

The treatment, which M. Chomel adopted, consisted in bleeding once and sometimes twice at the commencement of the disease, and in the use of demulcent drinks, emollient enemata, baths, and poultices to the abdomen. When the adynamic symptoms were "tres-prononcés," he gave Bourdeaux or Malaga wine freely; also quinine

---

* It deserves to be noticed, that in two cases, the pulse, instead of being accelerated, was actually lower than in health. In one, it seldom beat oftener than 48 times in the minute.

and other tonics. This treatment was quite necessary, when the vital powers were very low, the pulse feeble and slow, and the extremities cold. As long as the surface remained warm, and the pulse quiet, M. Chomel dissuades the use of stimulants.

*Intermittent Fevers.*—The number of cases admitted was 16; of which 13 were of idiopathic or essential, and 3 of symptomatic ague.* The greater number of cases occurred in the Spring and Summer months. In 10, the fever was of the quotidian, and in 3, of the tertian type. In 6 of the cases only, it was found necessary to administer the sulphate of quinine: in the other 7 cases, the disease ceased spontaneously. Hospital physicians are apt to fall into error, as to the medicinal value of certain remedies, from exhibiting them in cases, which might get well, without any physic at all. In the treatment of recent agues, it is judicious to leave the patient alone for two or three days on a regulated diet, before administering any medicines. M. Chomel is of opinion that salicine, digitalis, &c. which are highly praised by some of his brethren in Paris, are quite inefficient in confirmed intermittents. Of the three cases of symptomatic ague, in two of them there was gastro-enteric inflammation, and in the other one, there was a tubercular affection of the lungs.† In all of them the fever was of the quotidian type.

In two or three of the cases of essential intermittent, there was considerable enlargement of the spleen. The best remedy for this complication was found to be the quinine.

*Exanthemata.*—There were 8 cases of measles admitted: all recovered. The peculiar expectoration—opaque sputa floating in, or mixed with a turbid fluid*—was not so decidedly marked, nor was it so general, as had been remarked in other seasons. The rubeolar catarrh is as different from the common catarrh, as erysipelas of the skin is from the exanthema of measles. In one of the eight cases, there was no eruption of papulæ on the surface, although all the constitutional pathognomic symptoms —the running from the nostrils, the redness and weeping of the eyes, the hoarse resonant cough, and the pyrexia —were present.

Of scarlatina also, there were eight cases. In four only of them, was the peculiar papillated state of the tongue observed.

One patient, in whom the exanthematous disease was complicated with rheumatism, died. On dissection, the redness of the intestinal follicles—a lesion which has been generally observed in former seasons—was not present; but the mucous membrane at different parts "etait parsemée de taches hæmorrhagiques."

There were six cases of variola, and three of varioloid—in none, was there any symptom or occurrence worthy of particular notice.

Of nineteen cases of erysipelas, the phlegmasia affected the face in eighteen. All did well. Thirteen of the cases occurred during the Summer, and only six in the Winter months. In ten of

---

* This distinction of intermittent fever into essential, and symptomatic, is much insisted on by M. Chomel. In the former, there is no appreciable lesion or local distress, which can be properly regarded as the "point de depart" of the pyrexia; whereas in the latter form, there is an evident disorder of some of the viscera. "Antiperiodic" medicines are suited to the one from the very commencement of the fever, but not to the other set.

† Query.—Was this a case of genuine ague? We suspect not; but rather one of incipient hectic—which in truth is *intermittent* fever. We have oftener than once seen this mistake committed, and much mischief done by the endeavour to cure the supposed ague.—*Rev.*

* The reporter adds: "ce signe est tellement characteristique, qu'il suffirait à lui seul pour fair reconnaitre la rougeole." We much doubt the accuracy of this assertion.—*Rev.*

the cases, the cutaneous eruption was preceded by a painful swelling of the cervical glands; and M. Chomel, by attending to this circumstance, was sometimes able to predict the invasion of erysipelas. Eight of the patients had previously suffered from repeated attacks of the disease: one said he had had, during his life, at least thirty attacks.

The treatment consisted in bloodletting, purgatives by the mouth, and also in the form of enemata, and in the application of blisters or other revulsives to the lower extremities. In one case, in which the erysipelas was apyretic, bloodletting was not employed.

The phlegmasiæ of the digestive tube —stomatitis, angina, gastritis, and enteritis—did not exhibit any remarkable features. A few cases of dysentery were admitted: they were successfully treated with aperients.

There were nine cases of lead colic. Two of these presented certain anomalous characters. In one, the case of a man, who had been for a length of time exposed to the emanations from lead, there was the ordinary constipation, the retraction of the abdomen, the vomiting and the trembling of the limbs; but there was no abdominal pain:—it was an example of "colica pictonum sine colica."

In another case, there was amaurosis and tendency also to epileptic fits, added to the abdominal disease. The man was bled, and blistered on the nape of the neck; and then under the use of active purgatives, he quite recovered his health.

Among the phlegmasiæ of the respiratory organs, there were two well marked examples of the suffocative catarrh, which seem to consist in an intense form of bronchitis, affecting the whole extent of the air tubes in both lungs. One of these occurred in a middle-aged woman, who was admitted into the hospital on the fourth day of the disease. The dyspnœa was intense, the number of inspirations in a minute 44, the pulse was rapid, the face of a purple hue, and the anxiety dreadful. Bleeding and blistering were used, but without avail. She died asphyxiated. On dissection, the whole extent of the bronchial mucous membrane was of a deep red colour, (" une rougeur vineuse de toute la muqueuse qui tapisse les bronches,") and the lungs in certain parts were infiltrated with a bloody serum.

There were 14 cases of pleuritis. Of the four fatal cases the thoracic disease was, in three of them, complicated with meningitis, and in the remaining one, with pulmonary tubercles.

No fewer than 61 cases of pleuro-pneumonia were treated, and of this number, 14 died—certainly a great mortality; but be it noticed that some of the patients were "agonisans" at the period of their admission into the hospital, (in nine the disease was in the first stage—that of sanguineous engorgement or congestion—in 46, it was in the second stage—that of hepatisation—and in the remaining six, it had passed into the third stage—that of infiltration), and that in others, there was a complication of diseases. In four of the fatal cases, the pneumonia was complicated with meningitis. In eight cases (of which five were fatal), both lungs were inflamed at the same time; in 32, the disease was confined to the right, and in 21 to the left side of the chest. Very nearly the same proportion has been remarked in other clinical sessions.

In 22 of the cases, the upper lobe, or lobes of the lungs were chiefly affected. The danger of the attack was greatly influenced by the age of the patient: old people seldom recover.— With respect to the sex of the patient, 45 of them were males and 16 were females.

The diagnosis was in most cases sufficiently easy. Occasionally when all the rational or general symptoms of the disease—fever, pain in the side, cough, expectoration of rust-coloured sputa, &c. were well marked, the usual stethoscopic signs — dulness on percussion, and the crepitous rattle, and bronchial respiration heard on auscultation—were almost entirely absent. In such cases, the disease was probably seated in the deep parts of the lungs. The crepitant rale was sometimes observed to continue for several days, after the first stage of the inflammation had ceased, and even after the re-solution of the disease. The reporter conjectures that when this happens, there has probably succeeded to the state of sanguineous engorgement a slight œdematous affection of the pulmonic tissue, and that the persistence of the crepitation is owing to this cause.

That form of pneumonia, which is apt to occur towards the termination of organic diseases, and to prove very rapidly fatal, was observed several times in the course of the season.

We have already mentioned that in five cases the pneumonia was complicated with meningitis, and that such a complication is of extreme danger: the symptoms being obscure, the patient often delirious, and towards the termination becoming comatose.

In five cases the complicating disease was jaundice; three of these cases proved fatal. On dissection, there was no appreciable lesion of the liver.

With respect to the treatment* of the 61 cases of pneumonia—14 of which proved fatal—it consisted in " saignées, le tartre stibié à haute dose, les vesicatoires, et les bains."

*In every case, whatever was the stage or degree of the pneumonia*, bleeding from the arm was practised.

One only of the 9 cases, in which the inflammation was in its first stage, proved fatal; both lungs were affected at the same time. Of the 46 patients, in which the disease was in the stage of incipient hepatisation, seven died. The average quantity of blood drawn was two pounds and a half, or nearly three pounds. Every one of the remaining six cases—in which the disease was already in the third stage—proved fatal. Four of them were complicated with cerebral disease. In all the prognosis was most unfavourable from the first examination; nevertheless " on fit usage des saignées, de l'emetique et des vesicatoires!!" The reporter is candid enough to acknowledge, that in these cases the bleeding appeared to accelerate the death of the patients; " cette influence facheuse des emissions sanguines, meme moderées, (de dix onces à une livre) à été surtout evidente chez les deux malades, qui rejetaient des crachats jus de pruneaux, characteristiques de l'infiltration purulente du poumon, quoique le pouls presentât un certain degré de resistance qui semblait autoriser la phlebotomie."

It appears too that, in these six fatal cases, the tartar emetic in full doses had been given!

(It must be quite unnecessary to make any critical remark on the treatment which we have now mentioned. That any physician should dream of bleeding a patient, in whom pneumonia had advanced to the third stage, that of purulent infiltration, appears to us very wonderful; except indeed he is prompted by the laudable motive of putting a speedy termination to the sick man's sufferings.—*Rev.*)

Among the other thoracic diseases, there were two cases of gangrene of the lungs, twenty cases of phthisis, and four of pulmonary emphysema. Fourteen cases of acute rheumatism were admitted. In four cases only, were the sounds of the heart's action at all irregular. In one of these, occurring in a youth, who had lost four pounds of blood, there was a strong bruit de soufflet, which gradually abated and ceased. But it was doubtful whether this abnormal sound was attributable to any inflammatory affection of the heart, or merely to " une modification du liquide circulatoire" induced by the loss of blood. The latter interpretation is probably the correct one; as the same sound was very distinctly heard in a patient who lay inan adjoining bed, and who was labouring under peripneumonia which proved fatal; he had lost upwards of six pounds of blood, and on dissection, no traces of any disease of the heart were discoverable. In another of the four cases, in which the bruit de soufflet was heard, the patient died from endo-carditis, after having lost eight ounces of blood; all the affected joints were minutely examined

* We earnestly solicit the attention of our readers to this topic, as illustrative of the injudicious practice of even the moderate or "juste-milieu" section of the French school at the present time.—*Rev.*

on dissection, but not the slightest mark of morbid change was found in any of them. About the same time, a patient in the Hôtel Dieu, labouring under acute rheumatism, died from endocarditis, and in this case also the post mortem examination threw no light on the pathology of the articular disease; "les resultats de l'examen cadaverique ont été egalement nuls relativement au rhumatisme." With respect to the treatment of the rheumatic cases, which occurred during this clinical course of M. Chomel, it was remarked that very copious bloodletting had the effect of retarding the recovery of the patients. —*La Lançette Française.*

In the preceding report on the cases of pulmonic disease, we wish to draw the attention of the reader to the circumstance there stated; that in a considerable proportion of the fatal examples of peripneumonia, there was present a complication of cerebral disease, meningitis. The following observations may be usefully adduced here to illustrate this topic of pathology. They were published some time ago in a Belgian periodical, the Bulletin Medical Belge.

Some writers have of late years alluded to the not infrequent coincidence between affections of the lungs and encysted abscesses or other diseases of the encephalon. Professor Lallemand, in particular has insisted upon this topic, in his valuable work on the brain.

In the Gazette Medicale for September 1835, there is an interesting communication from the pen of Professor Albers of Bonn, containing the history of a case, where two circumscribed abscesses in the right hemisphere of the brain were found in a patient, who had been long affected with pulmonic disease. The following case is in many respects similar.

A soldier was admitted into the military hospital at Louvain, labouring under chronic pneumonia. He was gradually recovering from this disease, when very unexpectedly he was seized with violent convulsions of the right arm. These attacks returned very frequently, and were always accompanied with intense head-ache, and with an almost complete loss of consciousness, during the violence of the fits. On the following day the seizures were still more severe, and they had now extended to the right side of the trunk and to the right lower extremity. Bleeding was tried; but this was quite ineffectual. The actual cautery was then applied to the nape of the neck. This powerful revulsive remedy induced a decided amelioration of the symptoms for a short period; but they returned with aggravated violence, until the parts which had been the seat of the convulsions became affected with paralysis. The tongue was partially paralytic and hence the speech was rendered thick and confused. The patient died on the following day.

*Dissection.* On removing the calvarium, the inner surface of the dura mater was found at several points to be adhering strongly to the pia mater, which was in a thickened and opaque state. The cerebral substance of the middle lobe (it is not stated whether it was the right or the left) was softened and almost diffluent. At its upper part, an obscure fluctuation was perceptible, and on making an incision there, an encysted abscess, as large as a hen's egg, was laid open. The cyst itself was perfectly organized, and could be readily detached, without being lacerated. Its parietes appeared to be formed of two membranes, the outer of which was "blanchatre, de forme fibreuse," and the inner was softer, smooth on the surface and almost like a mucous membrane. Its contents were of a greenish colour, and of a very fetid odour. A small quantity of serosity was found within the lateral ventricles. The lungs presented numerous adhesions of the two pleuræ to each other. The parenchyma, and especially that of the left lobes, exhibited several nuclei of grey hepatisation.

In a recent number of the Bulletin Clinique de Paris, we find recorded the following case of serious encephalic disease, associated with pneumonia, and which had been unsuspected during the life of the patient. As a remark of general truth, it may be stated that all thoracic affections, when associated with symptoms of cerebral disturbance, ought to excite the alarm of the physician.

A middle-aged man, of apparently a sound constitution, was attacked with pneumonia. In addition to the usual symptoms, he suffered from an intense headache. He was bled freely and leeched. (It is to be noticed that this report commences on the sixth day of the illness). The headache was removed, but the chest distress became aggravated. He was again bled and died a few hours afterwards.

*Dissection.* Both lungs were found extensively diseased, from crude and softened tuberculous deposits, and from hepatization. The pia mater was infiltrated with an opaque fluid, and the two cerebral ventricles contained nearly two ounces of serosity.

*Remarks.* The reporter of this case observes that the patient seemed to have so sound a constitution, that the presence of tubercles could not have been reasonably predicted. The severe headache, which existed during the first six days of the attack, was no doubt dependent on the sub-acute inflammatory action of the cerebral meninges. The effusion was in all probability very rapid, (as there had been no stupor nor delirium except during the last few hours of life) and may be viewed as the cause of the sudden death.

---

### Illustrations of Thoracic Pathology—Connexion of Diseases of the Heart with Acute Rheumatism—Cases of Hydro-pericardium and Empyema — Piorry's Suggestion for treating Vomicæ.

### *Fatal Case of Acute Rheumatism—Appearances on Dissection.*

The following case will be read with much interest by every physician. It seldom happens that an opportunity of examining the pathological appearances in acute rheumatism presents itself.

A woman, aged 27 years, was admitted on the 21st March, 1836, into the Hôtel Dieu, under the care of M. Chomel, for acute rheumatism, which had lasted for the nine preceding days. The knees, hips, and loins were the parts chiefly affected. At this time there was no thoracic distress. She was bled to the amount of 18 oz. This operation was repeated on the following two days: the severe pains still continued. The knees had become greatly swollen, and the patellæ " sont soulevées par l'épanchement qui s'est formé dans les genoux." [This was probably a mistake. The swelling may have communicated to the finger a sense of fluctuation, although no effusion had taken place into the joint.—*Rev.*] The ankle and wrist-joints also had become severely affected with the rheumatism. She was again bled to thirty oz. At this period of the case, percussion on the præcordial region caused a slight degree of pain; but there was no dull sound elicited. The respiratory murmur was normal every where. The first sound of the heart was accompanied with " un tres leger souffle." The pains in the joints became so severe towards evening, that two grains of opium were administered.

Next day, the 24th, the patient was very weak and pale; the pains were stationary; the blowing bruit, attending the first sound of the heart, was stronger and more diffused. Saignée à 18 oz. The venæsection was repeated to the same amount on the 25th.

The patient became gradually weaker and more exhausted; the pains in the joints were still very severe, and they had now affected the elbows and shoulders; the pulse was rapid, soft, and occasionally intermittent; the number of respirations in a minute varied from 26 to 36; the blowing sound of the heart varied in intensity and also in extent at different times of the day; the respiratory murmur was perceptible over the whole chest, &c. Leeches, blisters, sinapisms, and low diet, constituted the treatment. On the 4th of April, when the patient had been incoherent and delirious during the preceding night, when the pulse was rapid and irregular, and when the number of respirations in a minute amounted to 44, thirteen ozs of blood were taken once more from the arm,* in consequence of a troublesome cough having come on, and a sibilant râle being heard over the right side of the chest. On the 9th of April, the day before her death, we are informed that the sounds of the heart were quite normal, that the pulse was 100 and regular, that the respiratory murmur was "un peu faible" in the lower part of the right side, and that the sound on percussion over that part was "un peu obscur;" and that the pains in the joints had ceased. A large blister was applied on the chest. next day the patient sunk, from exhaustion of all the vital powers.†

*Dissection.* All the joints, which had been affected with rheumatism, were carefully examined. The tumefaction had quite subsided. The synovial membranes were free from any traces of inflammatory blush, and the synovia was in moderate quantities, viscid, semi-transparent, of a yellowish colour, and inodorous. In short, all the component parts of these joints "ne nous offrent aucune altèration appreciable ni dans leur consistance, ni dans leur coloration, ni dans leur épaisseur." The pleura throughout was smooth and healthy. A pint of limpid serum, without any albuminous flocculi, was found in the right cavity. There was not any distinct trace of pneumonia, except in the lower third of the right lung, where the pulmonary tissue was dense and apparently impervious to air. The pericardium contained about two table-spoonfuls of a lemon-coloured serosity. The structure of the membrane was healthy throughout, except at one point half an inch square, which was covered with recently-effused lymph. On slitting open the cavities of the heart, the mitral valve exhibited numerous grey granulations, insulated or confluent, and varying in size from a pin's head to a millet-seed, and at one part there was "une veritable fausse membrane," which added considerable thickness to the valve; the chordæ tendineæ were enveloped in a soft, greyish, slightly adherent matter; the aortic valves were dotted with numerous granulations, similar to those on the mitral valve; the tricuspid valve exhibited a few, but seemingly of more recent deposit than those on the other valves.—*Journal Hebdomadaire.*

The preceding history will be read with great interest by the practical, as well as by the pathological physician. Fatal cases of rheumatism are exceedingly rare, and hence we have very few reports of the post-mortem appearances in the affected joints. In the present case, we are told that "l'examen de toutes les articulations n'a fait découvrir dans aucune d'elles le plus leger vestige d'un travail inflammatoire quelconque."

The treatment pursued was, in our opinion, most unwise and improper. Far too much blood was drawn, and the depleting regimen was continued at a time when the patient was ill able to bear it. There is no mention made of the use of mercury, colchicum, &c. Severe acute rheumatism can never be *safely* treated by the mere employment of vigorous depletion. This is a flagrant error in the practice of M. Bouillaud and some other physicians in Paris. We are surprised that M. Chomel should ever adopt it. The pleuritic and pericardial effusion were, we doubt not, the direct effect of exhaustion. We are expressly informed, in the report of the dissection—"l'épanchement sereux semblait être independant de toute phlogose, et ressemblait beaucoup à ces hydropisies pleurales, si fréquentes dans les affections organiques du cœur."

M. Bricheteau, in his recent lectures

* The reporter describes the appearance of the blood drawn thus:—"couenne n'existe que par places; caillot mou; la serositè forme les ¾ de la masse." In all the former bleedings the blood was buffy; the proportion of serum to crassamentum had gradually increased.

† It deserves notice, that at no period of this case had there been strong palpitations of the heart. M. Bouillaud regards the presence of these as one of the most constant symptoms of pericarditis.

at the Hôpital Necker, has approved of the practice of repeated bleedings, "coup sur coup," in acute rheumatism, as recommended by M. Bouillaud; but he stated that his confrere has surely exaggerated the frequency of the complication of pericarditis, or of endocarditis, with arthritic inflammation. M. Chomel also is of the same opinion. In his Clinique Medicale, he thus expresses himself. "M. Bouillaud has of late years maintained that pericarditis and endocarditis usually accompany acute rheumatism. This doctrine, although founded, it is alleged, on *facts*, has nevertheless been received with incredulity. In our own practice we have tested the accuracy of this announced pathological law, and have examined by auscultation and percussion all our rheumatic patients, but "deux fois seulement nous n'avons constaté dans le cœur d'autre derangement qu'un bruit anormal." M. Bricheteau very justly adds: "We ought not to forget that copious detractions of blood may of themselves induce the blowing, rasping, or rubbing sounds during the heart's movements, although no inflammation of any of the structures of the heart be present."

Many of the French journals have of late contained cases illustrative of the connexion of inflammation of the heart with acute rheumatism. We have frequently expressed our own sentiments on this subject, and deem it unnecessary to amplify at the present time. We shall therefore point our reader's attention to one only of the best papers, which we have met with in the French journals.

M. Cazaneuve on Endocarditis.

An elaborate memoir on endocarditis by M. Cazaneuve, of the Val de Grace Hospital, will be found in the Gazette Medicale for June 1836. He has minutely traced the literary history of this disease, which has of late excited much attention, from the period of the observations of Baillie and Portal down to the present time.

A striking example of it is related in the 5th vol. of the Edinburgh Medical and Surgical Journal. A young girl was, on the sudden recession of an acute rheumatic attack, seized with great thoracic distress. She died in the course of a few days; and on dissection, the internal surface of the right ventricle was found inflamed, and the tricuspid and mitral valves were coated with coagulable lymph. Kreysig has alluded to the coexistence of endocarditis with pericarditis, and to the frequent occurrence of both these forms in measles and scarlatina;* and M. Broussais in his Examen des Doctrines Med., has most ably pointed out the changes, which this disease is apt to induce in the substance, more especially in the valves of the heart, and has distinctly alluded to the occasional coincidence of inflammation of the lining membrane of the heart—to which he affixed the name of meningo-carditis—with acute rheumatism. Laennec appears to have been unwilling to recognise endocarditis, as one of the diseases of the heart. Bertin and Bouillaud in 1824 treat of it, as an affection "excessivement rare;" M. Andral in 1834 enumerated it as one of the causes of hypertrophy of the heart; M. Littré has treated of it in the Dict. Med. t. 9, and within the last two years M. Bouillaud has written a long treatise on it, in his Traité des Maladies du Cœur." To it we must refer our readers for particulars.†

---

* Kreysig very justly remarks that the tendency of the heart to be inflamed in scarlatina and measles accounts for the pulse often retaining its frequency and force in these diseases, when the other symptoms are yielding.

M. Tanchon (Journal Complement. t. 23) and Berard, (Dict. Med. art. Arterite) have alluded to the frequency of apparent inflammation of the lining membrane of the heart and of the aorta in fatal cases of variola.

† M. Bouillaud is certainly one of the most active and industrious physicians of the French metropolis; but

M. Cazaneuve, himself a disciple of the same school as M. Bouillaud, has detailed the particulars of five cases of alleged (we say "alleged," because the reported post-portem appearances have not quite satisfied us that there was genuine disease of the lining membrane of the heart—*Rev.*) endocarditis, and in not one of these cases had there been pre-existent rheumatism.

### Rare Case of Hydro-pneumato-Pericardium.

M. Bricheteau alludes to the greater frequency of heart-diseases among shoemakers than among any other class of artisans. He attributes this circumstance to the awkward and confined position of their bodies, when at work. The chest is necessarily compressed, and prevented from being freely dilated in any direction, either upwards, or from the sternum to the back, while at the same time the pectoral and other thoracic muscles are powerfully exercised. Their abodes too are almost always confined and unwholesome.

The following very interesting case occurred in a shoemaker, 59 years of age. In his youth, the patient had served in the army, and had, several years before, received a severe blow from the pole of a coach on the chest. From the date of this accident he had always experienced some thoracic inconvenience—dyspnœa, palpitations of the heart, &c.—which obliged him to sleep with the head raised high in bed. When the region of the heart was auscultated, M. Bricheteau heard distinctly a bruit of fluctuation, corresponding to each contraction of the heart. There was however no dull sound on percussion. The patient died, and on dissection the pericardium was found enormously distended, partly with a gaseous fluid, and partly with a purulent serum. The surface of the pericardium was lined almost throughout its whole extent with a fibrinous exudation.

---

he belongs to the "extreme gauche" party, *i. e.* the radical reformers in the medical republic of the present day. All his doctrines, and much of his practice too, is carried to a most extravagant "outrance." His opinions, as to the extreme frequency of endocarditis, and the necessity of "saignées formulées, ou coup sur coup," in inflammatory diseases, savours too much of the Sangrado school to meet with general adoption, at least in this country. —*Rev.*

### Case of Empyema—Paracentesis Thoracis—Recovery.

The patient, before he came under the care of M. Bricheteau, had been in the Hôpital de la Charité.

Succussion indicated very distinctly that the thorax contained a quantity of fluid, and also of air, which served as a means of transmission to the metallic tinkling sound which was heard. The serous effusion increasing so rapidly, as to threaten the suffocation of the patient, paracentesis thoracis was performed on the 21st of April, and gave issue to a large quantity of limpid serum, with great relief to the dyspnœa, &c. The operation was repeated on the 7th of August, when about 12 ounces of a whitish puriform fluid was discharged. The patient was greatly relieved for several weeks; but the metallic tinkling returned, and succussion again afforded proof of effused fluid, from the fluctuation which was audible. But both these symptoms became gradually less distinct, and the patient seemed to be recovering, when, in the month of December, an abscess formed over the place of the punctures. This was opened, and nearly half a pint of a yellow purulent fluid escaped. It could not be ascertained whether this abscess communicated or not with the cavity of the chest. Again it required opening in the beginning of March, and more pus mixed with coagula of blood was discharged. On introducing a probe, a large cyst was discovered. It was supposed that this cyst communicated with the chest, as the jet of the discharge appeared to be increased, whenever the patient coughed or inspired deeply.

Whether this supposition was correct or not, the progress of this curious case has been very satisfactory. The discharge has gradually ceased, the abscess healed up, the sounds of fluctuation and metallic tinkling are no longer perceptible, and we have reason to hope that the recovery will be permanent. The respiratory murmur however, and the sound elicited by the percussion of the right side are still much duller than in health.

### Compression of the Thorax recommended in the Treatment of Pulmonary Vomicæ.—Rarity of Phthisis among Coal-Miners.

M. Piorry, so favourably known by his works on thoracic diseases, acquaints us that, for several years past, he has been in the habit of compressing the chest in cases of excavations in the lungs, with the view of inducing a contraction and agglutination of their parietes.—That tubercular cavities do sometimes heal and become obliterated, cannot be denied by any pathologist, and that in such cases the part of the thoracic walls situated over such cavities usually becomes flattened and contracted, was asserted by Laennec, and has been verified by others; but that these fortunate changes should be effected, or even promoted by compressing the thorax, by means of graduated compresses over the suspected excavation, and of a belt or roller, appears to us *(Rev.)* to be of more than doubtful propriety. Are pulmonary vomicæ usually solitary? Certainly not. And even supposing that we could obtain the agglutination of one or of several of them, be it remembered that this alone will not be a cure of pulmonary consumption. It is a great error to regard this most formidable disease, as a mere malady of one part—the lungs. Those who have studied Louis' pathological researches, and who have read Dr. Clark's excellent Treatise, need not be told how widely diffused are the morbid changes throughout the system, when consumption has reached the stage of pulmonary excavation.

The treatment suggested by M. Piorry is founded on an erroneous and most hurtful doctrine—that the disease is a local one. He reports the histories of five or six cases, in which, having detected the existence of cavities in the lungs, he used compression of the chest; but, as might be expected, the remedy did not produce decided good in any of them. He talks, indeed, of "une amelioration marquée" in some of the cases; but such a vague account will not satisfy any rational physician.

With even more than a Frenchman's accustomed vanity, M. Piorry seems to anticipate important results from his proposed remedy, and with most philosophic bashfulness disclaims any personal merit; "parceque le succes devra etre attribué point à un medecin isolé, mais à la marche generale de l'esprit humain."

Should any of our readers wish to know more of our author's memoir, they will find it in the Bulletin Clinique for last May.

Dr. Valat, in a series of elaborate memoirs, published in the Revue Medicale, alludes to the great rarity of tuberculous diseases among the coal-miners at Decise in the department of the Nievre, where he has resided for many years. He has not met with one case of genuine tuberculous consumption, cases of which are so rife in all the hospitals of Paris, Lyons, and Montpelier; and yet pleurisies and pneumonias are by no means unfrequent. The observations of some English medical writers agree so far with those of Dr. V. that phthisis is certainly not a common disease among coal-miners. That they should be entirely exempt from it, we cannot expect.

---

### On the Pneumonia of old People. MM. Hourmann and Dechambre.

The following remarks on this important topic have been derived from a very extended series of observations, made chiefly in the practice at the great Hospice of the Salpetriere in which there are usually two or three thousand poor old women. MM. Hourmann and Dechambre have paid particular attention to the etiology of this affection, and the results of their enquiries are alike interesting and instructive.

The chief predisposing cause to the pneumonia in question is unquestionably to be found in the almost habitual bronchorrhea to which old people are subject. The mucous membrane of the bronchi is in a relaxed and weakened state; the secretion from its surface becomes more copious as we advance in life, and this indicates a tendency to congestion of its capillary vessels; the membrane itself is less sensitive, and the submucous muscular or fibrous tissue is less irritable than it was wont to be; the cartilaginous rings of the air tubes are more unyielding—all these circumstances operating together must predispose the lungs to be readily affected by atmospheric changes, and will account for the extreme obstinacy and frequent danger of such affections in old age. But there are other conditions of the system at this period of life, which act in a similar manner. Such for example is the frequency of heart diseases, and the narrowing of the thoracic cavity in consequence of the enlargement of some of the abdominal viscera.

Considering, therefore, the influence of these predisposing causes, it is not to be wondered at that pneumonic disease is of great frequency, and of most tedious obstinacy in old age; and the circumstance of both lungs being so much oftener affected simultaneously in aged people than in those of earlier life can only be explained on the plea, that both lobes have undergone certain unfavourable changes, by which they are more predisposed, than formerly, to be affected by morbid influences.

Of the occasional or existing causes of pneumonia in old people, by far the most frequent is the variableness of the weather. The influence of atmospheric conditions is well illustrated by the following table.

Of 156 cases observed in the Salpetriere during the course of one year,

| | | | |
|---|---|---|---|
| 17 | occurred in | January — | 6 deaths |
| 19 | ——— | February — | 14 — |
| 31 | ——— | March — | 20 — |
| 20 | ——— | April — | 9 — |
| 11 | ——— | May — | 3 — |
| 6 | ——— | June — | 4 — |
| 3 | ——— | July — | 2 — |
| *1 | ——— | August — | 1 — |
| 1 | ——— | September— | 0 — |
| 5 | ——— | October — | 4 — |
| 19 | ——— | November— | 15 — |
| 23 | ——— | December — | 10 — |

As a remark of very general truth, it may be stated, that the variableness of the weather is much more apt to induce pneumonia and bronchitis, than mere severity of the cold.

From the preceding table, it appears that the months of March and April are on the whole most unfavourable. The pernicious influence of sharp cutting winds has been noticed by all statistical writers. Hippocrates alludes to the frequency of chest disorders during the blowing of North-East winds. MM. Hourmann and Dechambre, confirm the truth of this observation.

"Ce que nous pouvons assurer relativement à nos vieillards, c'est que dans le cours des deux premieres années, et cette anneé meme (1836), au mois de Mars et Avril, pendant tout le temps (douze jours en 1834), qu'une augmentation considerable eut lieu dans le nombre des pneumonies, la fleche placée sur l'eglise de la Salpetriere a pris la direction du Nord-Est et s'y est maintenue invariablement. Nous avons dit qu' à cette epoque aussi la temperature etait abaissée et soumise à des variations."

The influence of long decubitus in bed ought certainly to be enumerated among, if not the inducing, at least the predisposing causes of pneumonia in

* During the months of August and September the cases were not so regularly registered as during the other months. There may have been therefore admitted two or three cases more, than we have details of.

old people. Hitherto the influence of this circumstance has not been sufficiently attended to by medical enquirers. That the statis of the blood, in those parts of organs which are kept for a length of time in one position, has a tendency to induce, or at least to favor the accession of a troublesome sort of inflammatory action, is strikingly illustrated in the case of old people, who have been long bed-ridden.

From the researches of M. M. Hourmann and Dechambre, it appears that the posterior and inferior parts of the pulmonary lobes are much more commonly affected with *intervesicular* pneumonia than the anterior and upper parts; that *vesicular* pneumonia is more frequent in the anterior than in the posterior edges of the pulmonary lobes; and that pneumonia of the superior lobes has in most cases its seat all around the fissure, which separates them from the middle lobes, and pneumonia of the lower lobes is very generally seated in their posterior parts. This predilection of pneumonia for those parts which are most "declives" can be fairly attributed only to the mechanical accumulation of the blood.

Our authors state also that pneumonia of the lower lobes is much more frequent on the right than on the left side, and account for this by saying that most bed-ridden people usually lie most on the former side.

The influence of prolonged decubitus in determining the seat of pneumonia in old people has been much insisted upon and very ingeniously discussed by M. Piorry in his memoir on what he calls "pneumonic hypostatique."*

To sum up in a few words the doctrines of M. M. Hourmann and Dechambre on the etiology of senile pneumonia, it may be stated, that some of the causes, as for example weakness of the system, and tendency to chronic

* There is a notice of M. Piorry's memoir in the number of this Review for July 1833. As the subject is but little known, we shall make one or two extracts from it. "It is well known to anatomists, that the posterior parts of the lungs are almost always found on dissection to have a deep purple venous colour, while the fore-parts of the lobes are pale and speckled. This appearance of congestion is correctly attributed to the subsidence of the blood after death towards the most depending parts, and constitutes the "engouement cadaverique" of the French pathologists. Now M. Piorry is of opinion that in enfeebled states of the system, especially in aged people, a tendency to the the same subsidence may take place during life, when the patients are long confined to the horizontal posture, and may thus, if it does not actually prove an exciting cause, at least must aggravate any inflammatory affection of the lungs. Eight illustrative cases are adduced; they occurred in old people admitted into the Salpetriere as objects of charity.

Professor Boyer long ago remarked that it was most injudicious to confine old people for any great length of time to bed; and even, when it is absolutely necessary, as after fractures, or other severe injuries, they should be told to sit up occasionally in bed, in order to prevent the congestion of blood, not only in the skin of the back, but also in the depending parts of the internal viscera." p. 212.

The following notice of the sentiments of another estimable French physician may be appropriately introduced here.

M. Bricheteau has of late met with several cases of fatal pneumonia, characterised on dissection by a ramollissement of the pulmonary tissue, which he calls "splenification," from its resemblance to softened spleen. In all the cases, there had been a considerable impediment to the free circulation of blood through the lungs, in consequence generally of an associated heart disease. The tissue had become as it were soaked with blood, and so much softened, that it was easily lacerable, and reducible into a soft pap on pressure.

This sort of pneumonia is much more frequently met with in the old and enfeebled, than in younger patients; and is generally associated with disease of the left side of the heart.

catarrh, operate on all the patients; that other causes, as diseases of the heart, are present in a large number; and lastly that those patients, who are bed-ridden, are on the whole more susceptible of being affected by atmospheric vicissitudes, than those who are able to be moving about.

The accession of senile pneumonia may take place in two very distinct forms. It is either sudden, setting in with a chill, pain of the side, as is ordinarily the case in young subjects; or it may creep on insidiously, and establish itself, before its existence has been suspected, and without the presence of any well marked symptoms. At the Salpetriere, senile pneumonia assumes almost always the first of these fevers during the months of March and April. The shivering and the attack of pain are often preceded for several days by headache, general languor, coryza with or without epistaxis, flying pains in the limbs, &c. and the pneumonia being once fairly established, the patients soon fall into a state of stupor and adynamia.

In the second mode of attack, there is neither shivering, nor any pain in the side at first: the patient only feels ill at ease, and altogether more feeble than before; the breathing is hurried and sometimes irregular; there is a paroxysmal cough, accompanied with difficulty in expectorating the phlegm, the skin is warm, and the tongue is dry. Such are often the premonitory symptoms of pneumonia in old people. But even these, now enumerated, are not always present. There may be neither cough, nor any disturbance of the breathing, even although the patient had been, before the attack, subject to an asthma, or chronic catarrh, and the only symptoms may be a general malaise and feebleness. This most unexpected occurrence has been repeatedly noticed by M. M. Hourmann and Dechambre, and from its very singularity, it becomes a valuable symptom to the experienced practitioner. In reference to the dyspnœa, our authors very properly inculcate on their readers the propriety of not trusting to the mere accounts of the patient. The physician ought to examine the breathing for himself, to count the number of respirations, to percuss the chest, and to ascertain the sounds elicited on auscultation. With all the assistance to be derived even from these means of exploration, it is occasionally not easy to form an accurate diagnosis of the case, and to prognosticate its probable result. Take for example such a case as the following. One of the old inmates of the Salpetriere rises at her usual hour, makes her own bed, eats her food as usual and moves about the ward; in the course of the day she feels fatigued and exhausted, lays down on her bed, and, after a short struggle of difficulty in breathing, expires. "C'est la une des *morts* dites *subites* de vieillesse à la Salpetriere." On dissection, the physician is much surprised to find a large portion of the pulmonary parenchyma in a state of suppuration. Some very remarkable cases of this description have been observed by M. M. Hourmann and Dechambre in old women admitted into the Infirmary of the Salpetriere for other diseases. For example, a poor Vieille, 85 years old, had been admitted a few days before for a stomach complaint: she was quite gay and cheerful, and to shew her good appetite to the doctors, she eat a piece of hard biscuit in their presence.

The visit of the infirmary was not over, when one of the nurses came to inform them that their old patient was dying. They arrived just in time to see her expire; some of the biscuit crumbs still hung around her mouth. On dissection, the entire substance of the lower lobe of the right lung was found in a state of free suppuration. M. Falret mentioned a similar case to the authors. An old insane woman, remarkable for her habitual loquacity and strength of voice, very suddenly fell down and died in the course of an hour or so. The whole extent of the right lung was found in a state of grey hepatisation.

A very important conclusion, which M.M. Hourmann and Dechambre have deduced from their researches, is, that senile pneumonia very generally commences and advances in an obscure latent form, when there is co-existent cardiac or cephalic disease—very common complications—and that on the other hand the attack is usually sudden and acute, in persons who are free from such diseases.

The *symptomatology* of senile pneumonia may be, in part at least, inferred from the preceding remark. The pain is seldom sharp or severe; and this is often the case, even when the corresponding portion of the pleura is inflamed at the same time. The patient complains of a mere sense of soreness or uneasiness over the entire affected side. In those cases, in which the disease commenced with a stitch, this seldom lasts above two or three days, and then it is replaced by a diffused obtuse pain. The dyspnœa is often quite as unsatisfactory a symptom as the local pain. The breathing may be not at all embarrassed; usually it is accelerated; but in some cases it is even slower than usual. It has been observed by our authors, that the dyspnœa is always much less severe when the disease affects chiefly the lower lobes. When the upper lobe or lobes are inflamed, the breathing is almost invariably much distressed, and often constant orthopnœa is present. Occasionally however the inflammation of the upper lobes commences in a latent form, and all the symptoms remain, throughout, obscure and indistinct.

What we have said of the pain and dyspnœa, may be extended to the cough, as one of the symptoms of senile pneumonia. It is sometimes so inconsiderable, that the patient scarcely is aware of its presence.

He has perhaps only two or three fits in the course of the day; and these are apt to come on, only when he makes any exertion, or changes the temperature in which he is in. The expectoration also in not a few examples affords no evidence of the existing disease.

We have already remarked that M.M. Hourmann and Dechambre have repeatedly observed that in those cases, where the patients had been subject to a chronic catarrh before the accession of the pneumonia, the expectoration often ceases altogether.

With respect to the auscultatory symptoms of senile pneumonia, they are not, on the whole, so distinct and satisfactory as in the pneumonia of younger people. The genuine râle crepitant is rarely to be heard; mucous and sonorous râles are the predominating sounds. Sometimes the râle has an unusually dry resonance, and resembles the crackling of parchment rubbed between the fingers. The respiratory bruit is almost always more or less bronchial in the neighbourhood of the affected parts. The complete extinction of the respiration over the parts themselves is a much more rare occurrence in the old, than in the young and middle-aged, affected with pneumonia.

It particularly deserves to be noticed that, in some cases of senile pneumonia, auscultation affords no evidence of its existence. The words of our authors are :—"Nous avons vu des pneumonies, chez nos vieillarde, traverser toutes leurs periodes, sans donner lieu à aucun râle. Alors ou bien l'on n'entendait rien, ou bien le bruit respiratoire etait tres fort et pure. Nous avons recueilli un fait de ce genre, meme dans un cas de pneumonie de tout le lobe superieur droit, et l'auscultation a été pratiquée jusqu'à l'instant de la mort."

During the progress of senile pneumonia, the functions of the cerebrum are very generally disturbed; the patient becomes delirious, and the delirium lapses more or less speedily into coma. The period, at which the mental disturbance usually commences, is when the process of suppuration is about to be established; sometimes, however, the invasion is much earlier. In the greater number of cases, there is no delirium nor coma; but only decay and hebetude of all the intellect and moral powers. Without being positively irrational, the patients appear to be quite lost; they do not understand what is said to them; their answers have, perhaps, no reference to the questions put, and they remain taciturn and take no notice of any thing. In short, they lapse into that condition so emphatically described by Shakespeare as "second childishness and mere oblivion."

The symptoms afforded by the state of the tongue, of the skin, the bowels, and the urine, are far too uncertain and unsteady to warrant any confidence being placed in them. The pulse itself is apt to misguide us, if we attend too much to its state. That the very essence, if we may so speak, or the proximate cause of the disease in old persons, is different from that of pneumonia in younger subjects, is rendered probable by the circumstance of the blood, when drawn in the former set of cases, being generally without any buffy coat. Of 24 venæsections, practised by MM. Hourmann and Dechambre, the blood was buffy eight times only; and in 23 other cases, the blood was buffy in nine only of them. Of 17 cases, in which the blood was found to be buffy, the invasion of the disease had been acute in 15, and latent in two. Of the 30 cases in which it was not buffy, the invasion had been latent in 11, and acute in 18 of them.

The physical characters of the coagulum are, in general, very different from those exhibited by buffy blood. It is not red and firm, but soft, black, or even of a greenish hue. Sometimes the blood does not form a proper clot, but assumes the consistence of molasses.

MM. Hourmann and Dechambre very properly remind their readers of these facts, when they come to treat of the appropriate remedies for senile pneumonia. Free bloodletting must be practised with great circumspection; the powers of life are easily made prostrate, and if once permitted to be so, there is little hope of re-animating them. They tell us that they have sometimes found it necessary to exhibit wine and other stimulants, at the very time when they were drawing blood. Perhaps, under such circumstances, it would be more judicious to employ local depletions, by cupping, than general evacuations.

Emetics are highly praised by our authors. They have no faith in the contra-stimulant practice of exhibiting large doses of tartar-emetic. Blisters, large and applied directly to the chest, are among the most efficacious remedies: in most cases, they may be used with advantage from the very commencement of the disease. [No allusion is made to the use of expectorants: the judicious use of oxymel of squills, compound tincture of camphor and syrup of poppies, in the form of a mixture, will be found of sovereign benefit in most cases.—REV.]—*Archives Générales de Médecine.*

---

REMARKS ON INFANTINE ASTHMA, CONNECTED WITH AN ENLARGED STATE OF THE THYMUS GLAND, WITH CASES.

This variety of asthma, which we take to be nearly the same as the "acute asthma" of Millar, and the "spasmodic croup" of Guersent, Michaelis, the "laryngismus stridulus" of Good, Ley, &c. and to which the German writers have given the appellation of "asthma thymicum"—from its presumed cause, the enlargement of the thymus gland—is more common during the first, than during any subsequent year of a child's life. The little patient, who perhaps had been in perfect health the minute before, is suddenly seized with a complete stoppage, or a most painful difficulty, of breathing, and the body and limbs become affected with violent convulsions. If the breathing continues, each act of inspiration and expiration is attended with a whistling or crowing sound, as if the air had to pass through a narrow metallic opening. This frightful state will last for one, two, or three minutes. Sometimes the child recovers from it with a scream, and the breathing resumes its healthy regularity; at other times the recovery is more gradual, and the child remains for a considerable period exhausted and drowsy.

These attacks are apt to return at irregular intervals. They occur most frequently on awaking from sleep, or when the child is fretful or has been crying. In short, any change in the act of respiration predisposes to the coming on of the suffocative paroxysm. At first the attacks are rare; but gradually they become more frequent; and in the worst cases, ten and even twenty paroxysms have been known to occur in 24 hours. Often the child dies asphyxiated in the course of a minute or two; or, if it survives this period, convulsions are apt to ensue, and then the death seems to be more of an apoplectic character. Numerous dissections have shewn that, in addition to the post-mortem appearances usually observed in cases of asphyxia, there is almost always an enlarged state of the thymus gland. Sometimes this organ is found so much increased in dimensions, that it has pushed back the lungs, and compressed the trachea and the large blood-vessels at the root of the neck. The structure of the gland is generally healthy, and when cut into, a milky fluid flows out.

The children most liable to this form of asthma are those of a strumous constitution, and the predisposition to it is strongest after attacks of catarrh, croup, hooping-cough, or measles, or, in short, of any disease in which the breathing has been much affected. Dentition also, and all disturbances of the stomach and bowels, are apt to induce it.

The treatment necessarily resolves itself into the management of the suffocative paroxysm, and into the arresting of its disposition to return. Unfortunately, not much can be done during the paroxysm, and we have known many cases where the patients perished on the first attack. The infant should be held up, with its head inclined rather backwards, strong smelling salts should be applied to the nostrils, water sprinkled on the face, and the back well rubbed, and tapped every now and then smartly with the hand.

During the intervals—and it deserves notice that the health of the child often appears to be completely restored, when the paroxysm has ceased—small doses of musk, assafœtida, zinc, especially the hydrocyanate, in cherry-laurel water, should be diligently administered for a length of time. Occasionally, purgatives also are necessary. Leeches and blisters over the seat of the thymus gland may be advantageously used. M. Fingerhuth, who has published an able memoir on Thymic asthma in Caspar's Wochenschrift for 1835, recommends the employment of iodine baths, and of frictions, over the seat of the thymus gland, with hydriodate of mercury ointment.

*Case* 1. A child, when five months old, suffered from an attack of bronchitis, which left a tendency to attacks of suffocative asthma, whenever he awoke from sleep. The fits at first were not very severe; but gradually they became more aggravated, so that sometimes the breathing was completely suspended, and then the body was convulsed, stiff, and bent backwards. Each paroxysm, after lasting for one or two minutes, terminated by a fit of shrill, interrupted crying, and the child was soon as cheerful and well as before the attack. In one of these fits, the child died asphyxiated.

On dissection, both lobes of the thymus gland were found to be considerably larger than usual, occupying the whole of the anterior mediastinum; and a middle portion, situated between the two lobes, rested upon, and appeared to compress the common jugular vein. The gland was of a firm consistence, and weighed about ten drachms.

*Case* 2. A scrofulous child, four months old, had frequent attacks of oppression in breathing, and general disquietude, which were attributed to a disordered stomach, and were treated with emetics. Two months afterwards, when Dr. Hirsch first visited this child, the paroxysms of asthma had increased greatly in frequency and in severity. When the attack was coming on, the child threw his body convulsively back, and made one or two efforts to draw in a breath; and as it began to yield, he made five or six shrill inspirations, which were not followed by expirations; the body lost its spasmodic stiffness; and after a short groaning and crying, he seemed to be almost quite well. Besides these attacks of asthma, this child was frequently seized with general convulsions of the body and limbs; the thumbs were forcibly bent in upon the palms; the whites of the eyes were rolled up, and a quantity of frothy saliva collected about the lips. Death took place on the second day after Dr. Hirsch's visit. On dissection, the thymus gland was found much hypertrophied: it extended from the thyroid gland above to the pericardium below, and adhered intimately to the arteria innominata and left carotid arteries.—*Schmidt's Jahrbucher der In-und Auslandisch: Medicin.*

*Remarks.* It appears to be quite a favorite doctrine among the German, and also, we believe, the Danish physicians, to refer the paroxysmal asthma of infants to a disease of the thymus gland. There is an interesting paper on the anatomy, functions, &c. of this organ in the 17th vol. of this Journal, p. 139, which will repay the reader's studious perusal. We were, we believe, the first to introduce this pathological subject to the notice of the profession in this country.—*Rev.*

---

### On the Nature and Seat of Angina Pectoris.

Dr. Heberden, in 1768, was the first who accurately described this most distressing malady,* and distinguished it from other thoracic affections, of which it had been supposed to be merely an occasional accompaniment.

Since his time, numerous contributions have been made by various authors to the history of its symptoms, causes, and treatment. Very considerable difference of opinion however has prevailed, as to the real nature and seat of the distress; owing, no doubt, to the error so frequently committed by medical men, of attending exclusively to the results of their own observation and experience, without regard to the recorded observation and experience of others, and also of deducing general conclusions from a too limited number of facts, however correctly these facts have been ascertained. The disease is not a very common one; a physician therefore has seldom the opportunity of examining many cases; and moreover the morbid appearances, which have been found in such as have proved fatal, are by no means uniform, or even similar, in all. The characteristic symptom of angina pectoris is a severe pain, felt immediately behind the sternum, and accompanied with a sense of strangling and dreadful constriction of the chest, which prevents the patient from drawing in a deep or easy breath. The pain is usually seated rather more on the left than on the right side, and it generally extends some way up the neck and along the arms, especially the left one, as far as the elbow, or wrist-joints. While the pain lasts, the pulsations of the heart are confused and irregular; sometimes violent and tumultuous; at other times extremely feeble, or even scarcely perceptible; and the patient is threatened with constant syncope: the breathing is hurried, short, and often so imperfect, that the movements of the chest are hardly to be perceived.

The attack of pain, (or, as it has been recently denominated, sternalgia,) is usually sudden and unlooked for; the patient being perhaps seized in a moment, having had little or no warning of its approach. In numerous cases the paroxysm is preceded by derangement of the stomach and bowels, indicated usually by flatulence, acid or unpleasant eructations, constipation, pains in the limbs, amounting sometimes to severe cramps. Bodily exercise, the impression of cold air on the surface, and strong emotions of the mind, are the most common exciting causes; but as the disease advances, the paroxysms sometimes occur even during sleep, or at other times when the patient is perfectly quiet. The attacks are more frequently observed in the evening than at

---

* It has been conjectured that the disease of which Seneca died, and which he himself calls "suspirium," was angina pectoris.

It is singular that Cullen has not noticed the disease in his Nosology. He surely must have seen several cases of it in his extensive practice; but may have very possibly mistaken them for asthma, or hydrothorax.

any other period of the day. This may probably be attributed to the stomach being more liable to be deranged by dinner, than by any other meal. So dreadful is the agony of the pain, that the patient is stricken mute and helpless, and is forced immediately to discontinue whatever he was doing before its seizure. A certain degree of relief is often obtained by pressing the chest against any hard object, or by having an attendant to beat and thump it, and also the back, firmly with his fist. As the fit declines, a quantity of wind is usually discharged from the stomach, and sometimes the other contents of the stomach are rejected by vomiting.

In the early stages of the disease, the attacks usually last only for a few minutes; but as it advances, they become much more lengthened, continuing from half an hour to an hour and upwards. During the intervals, the health of the patient often appears to be quite restored.

Angina pectoris is of much more frequent occurrence in the male, than in the female subject. It is more common between the fiftieth and sixtieth years, than at any other period of life.* Men of sedentary habits, when disposed to corpulency, and to rheumatic or gouty affections, and especially if they have suffered much from mental distress, are those who are chiefly liable to attacks of this disease. Heberden, Macbride, and Darwin, were of opinion that angina pectoris is essentially a spasmodic or nervous disease; and Desportes and Jurine of Geneva referred the spasm to an affection of the pulmonary and cardiac plexuses of nerves. That the disease is, in some measure, truly of a nervous character appears from the suddenness of its attacks, from the short duration of these, from their being very often followed by a state of apparent health, from the influence of mental emotions as an exciting cause, and from the want of uniformity in the necroscopic appearances in fatal cases. But we should commit a grievous error, if we were to suppose that all organic and appreciable lesions of the respiratory and circulatory organs were very generally absent. The paroxysmal character of the disease is not, we must remember, sufficient evidence of its purely spasmodic character; for asthma, and numerous hepatic and nephritic maladies, often exhibit the same feature during the life of the patients, and yet extensive degenerations of structure may be found on dissection. The circumstance too, of females and young people being so much less subject to angina pectoris than middle-aged males, may lead us to suspect the truth of its being of a purely nervous character. With respect to the doctrine of those physicians who refer the disease to an essential lesion of the thoracic, the pulmonary, and cardiac nerves, it is unnecessary to canvass its correctness; as it has not been supported by any pathological proofs.*

* Dr. Copland states that, of nearly one hundred cases, about seventy occurred in persons upwards of fifty years of age; and seventy-nine out of the number were males. It is equally common in persons of a spare as of a full habit.—*Rev.*

* This doctrine, although it is summarily rejected by our author, has been supported by some of the highest authorities on this disease, and appears to us to be, perhaps, the most satisfactory. Laennec was of opinion that angina pectoris is usually dependent upon an affection of the pneumogastric, or of the cardiac, or both sets of nerves. He adds:—"The anterior thoracic nerves, originating in the superficial cervical plexus, are moreover frequently implicated; and this is sometimes further the case with the branches derived from the lumbar and sacral plexuses, when the thigh and leg participate in the attack, which occasionally happens."—Dr. Chapman, in a very able paper published in the seventh volume of the American Journal of Medical Sciences, expresses similar sentiments: "That the disease is a species of neuralgia, I am entirely persuaded, commencing for the most part in the pneumogastric nerve, and spreading in different direc-

Influenced by the attentive examination of a few cases, several authors of respectability have pronounced that the usual cause of angina pectoris is a metastasis of gouty disease to the heart, lungs, or diaphragm. This opinion, however, is too vague to warrant our unqualified assent; for in a multitude of instances, no gouty disposition can be discovered. Other authors have supposed that angina pectoris is invariably dependent upon some fixed organic disease of the thoracic viscera. Drs. Jenner and Parry have, it is well known, particularly insisted upon the ossification of the coronary arteries of the heart, as the most frequent lesion: but this doctrine is very far from being universally, or perhaps even generally, correct. Various other morbid lesions of the heart, such as hardening of the semilunar and auriculo-ventricular valves, excessive accumulation of fat about the heart, softening and paleness of its substance, &c. have been particularized by Drs. Wall, Fothergill, and a host of occasional writers. It is now generally admitted that, all such organic diseases are only of casual occurrence in cases of angina pectoris; and that, on the other hand, they are not unfrequently discovered after death in the bodies of those, who have not during life suffered from this disease. Many of the Italian physicians have supposed that the most frequent cause of angina is the pressure, which a greatly enlarged liver or other abdominal viscus may exert upon the heart, by inducing a certain degree of paralysis of its muscular texture. But surely this is a most improbable explanation; for, not to allude to the circumstance of the two viscera, (when we talk of the liver and heart,) being situated on opposite sides of the body, may we not enquire how comes it, that angina is so rare a disease, considering the very great frequency of hepatic lesions? The attention of Dr. Gintrac, the author of the present memoir, was first specially directed to the pathology of angina, by a case which he attended in the year 1824. He was particularly struck with the exact seat, in every paroxysm, of the excruciating pain; this he invariably found to be immediately over the rising arch of the aorta. There were not any marked symptoms indicative of an organic disease of the heart, or of the lungs; all the sufferings appearing to be referred to the place, we have mentioned. The circumstance too of the arterial pulsations being strong and regular, taken in connexion with the uniform seat of the pain, led Dr. G. to suspect that, the disease belonged rather to the aorta, than to the heart itself. Numerous other considerations tended to confirm him in the correctness of this opinion. Certain it is that, in most fatal cases of angina some appreciable morbid alteration is discoverable either in the aorta itself, or in its semilunar valves, or in the cardiac arteries, or perhaps in all these parts at the same time; parts, it is to be noticed, all appertaining to the same class of structures. They may therefore be considered as only different portions of one organ; and hence we are led, a priori, to anticipate a similitude in their morbid changes. From a studious investigation of most of the cases on record, as well as from an attentive examination of several cases which have occurred in his own practice, Dr. Gintrac has been led to believe that the primary seat of angina pectoris is always in the aorta itself, or in some of its appendages. The most common lesions are dilatation of its tube, with thickening, and cartilaginous or osseous induration of its structure. In some cases, Dr. Parry and others have noticed visible traces of inflammation on the outer surface of the aorta, and its vasa vasorum have

---

tions, as other nerves may become involved. The derangement of the heart and other structures, with which it is sometimes associated, I hold to be coincidences or effects, and not the cause; since, among other reasons which might be adduced in corroboration of it, the disease has undoubtedly prevailed independently of such organic lesions, and conversely these have existed without occasioning it. My conviction is, that the immediate cause of the neuralgia is derived from irregular gout."
—*Rev.*

been found, by other observers, highly injected. In several instances, its lining membrane has been of a deep red colour, and exhibited numerous rugosities, and minute ulcerations scattered here and there. Occasionally these patches of superficial ulceration are of considerable extent. Often the aorta alone is found diseased: but, not unfrequently, its appendages also are similarly affected. Thus its valves may be thickened, warty and ossified, and the coronary arteries may have become cartilaginous or osseous. In the case of the late celebrated John Hunter, and in other examples related by Drs. Fothergill and Parry, the carotid arteries have been discovered to be ossified, along more or less of their extent. This coincidence of disease in the aorta, and in the arteries which are first given off by it, deserves notice; as it shows, that it is in this "ensemble" of parts, connected with each other by a similitude and indeed continuity of formation, that the seat of angina pectoris very generally exists. Although the lesions of the aorta and of its appendages may appear at first sight to be somewhat different from each other, they are all referrible to the same general cause, viz. chronic inflammation. The operation of this diseased action is at once obvious and indisputable, in such cases as exhibit these parts injected, thickened and ulcerated; and M. M. Bertin and Bouillaud have shewn that, dilatation also of the aorta is often primarily dependent on an inflammatory irritation of its parietes.

It may be objected to the doctrine of angina pectoris being the effect of chronic inflammation of the aorta and its appendages, that numerous cases of this disease have been recorded, in which these parts were found on dissection quite exempt from any appreciable morbid change.

Dr. Gintrac replies to this objection that, he does not mean to advance his doctrine as uniformly or exclusively, but only as very generally true; and suggests that, if the cases, apparently contradictory of its truth, be analyzed, we shall find that they are less opposed to his views, than may at first sight be imagined. The state of the lining membrane of the aorta and of its branches has been often overlooked by pathologists.—*Bulletin Medical Belge.*

It appears to us that Dr. Gintrac is much more successful in exposing the errors of others, than in establishing the correctness of his own opinions. We cannot concede to him, that angina pectoris is generally, far less invariably, connected with disease of the aorta or of its appendages. We have already stated that, in numerous cases, it appears to us to be of a strictly neuralgic character. The symptomatology, as well as the therapeutic history, of the disease lead to this conclusion.—*Rev.*

---

### On the Cause of the First Act of Respiration in the New Born Infant.

Prochaska attributed the first respiratory movement of the infant to an instinctive impulse. But instinct is dependent upon the brain; and, in Meckel's work on Pathological Anatomy, we read of a multitude of instances of anencephalic children breathing regularly for a limited time.

Blumenbach refers the first act of respiration to several co-operating agencies; such as, to congestion in the aorta induced by the obstruction of the umbilical arteries, to the danger of strangulation from the interrupted oxygenation of the blood, to the impression of the atmospheric air on the cutaneous surface, &c. All these influences must certainly tend to excite various movements, and more especially those of the dilatation of the thorax, and may thus cause the drawing in of the air to fill up the vacuum. But, apart from the doubts which the first two points may reasonably excite, the theory of Blumenbach supposes, on the part of the infant, a feeling connected with consciousness;—a presumption, which is controverted by the well ascertained fact of anencephalous monsters being capable of respiration. Rudolphi, in his Elements of Physiology, has proposed the following explanation. The muscles of the face are called into action by the unpleasant or painful impressions, to which the child is exposed during and immediately after delivery; the mouth and nostrils are thus opened and dilated, and the air is involuntarily drawn in. It will be observed that, Rudolphi calls into requisition Sir C. Bell's beautiful theory of the concatenation or associated sympathy of the muscles of the face with those of respiration.

Müller ascribes the breathing of the new-born infant to the stimulus or irritation, which the blood, immediately oxydising itself, or about to be oxidated in the lungs (das in den lungen sogleich sich oxydirende blut), produces on the brain and medulla oblongata.

But to this theory it may be answered, that the air, although by its elasticity it may be admitted into the trachea and its larger branches, cannot well force itself into the pulmonary cells (in which alone the oxidation of the blood is affected), because they are closed and fallen together (zusammengefallen). And moreover, what good reason is there for supposing that the nerves from the medulla oblongata require oxygenized blood to excite them into action; seeing that other nerves, those derived from the spinal marrow, possessed ample activity during intra-uterine life; as is satisfactorily proved by the movements of the body and limbs before birth.

The author seeks for another interpretation, derived in part from the researches of Dr. Marshall Hall on what he has denominated the reflex functions of the nervous system. It is well known that, irritations of all parts, which are invested with mucous membranes, have a tendency to excite the movements of the respiratory apparatus. Irritation of the nostrils induces sneezing; irritation of the throat and stomach induces the efforts of vomiting; irritation of the lower bowels, of the bladder and urethra, of the uterus and vagina, causes a sympathetic action of the diaphragm, abdominal muscles and of other parts involved in the act of breathing, and thus gives rise to the distressing symptoms of tenesmus, ischuria, &c.; and lastly irritation of any part of the air-passages at once induces vehement coughing.

To a certain extent, similar effects are produced by irritation of the cutaneous surface, as appears from the following considerations. Tickling certain parts of the skin excites immediately irrestrainable laughter; the application of sudden cold is followed by a succession of involuntary interrupted inspirations; —hence the utility of sprinkling cold water on the face in syncope—infants, apparently still-born, may be often made to breathe by a few smart strokes on the back; bodily suffering, and more especially that arising from any cutaneous injury, as from wounds, burns, &c. excites crying and sometimes convulsive laughter; and lastly, any decided impression on the surface of the body has a marked effect in abating a paroxysm of asthma. As then the surface of the infant is subjected to considerable irritation, from the pressure and uneasy position experienced during delivery, and also from the impression of the cool atmosphere, when it is born, we may perhaps most rationally explain the occurrence of the first act of breathing, by appealing to the reflex operation of the cutaneous on the respiratory nerves. —*Kleinert's Allgeneines Repertorium.*

The problem, involved in the preceding observations, is not very easily solved to our satisfaction. It seems probable that, the changes induced in the state of the circulation of the blood in the heart and great vessels, in consequence of the interruption, more or less complete, of the flow through the umbilical cord, excites a certain uneasy feeling (similar to that experienced when the breath has been held in for some time) within the thorax; and this prompts the animal to dilate it, and thus to give admission to the external air into the lungs. The fact however of the fœtus sometimes emitting distinct cries in utero—a well-marked case, of which anomaly is narrated in our Number for last July, vide Review of Dr. Collins on Midwifery—throws considerable doubt on the correctness of any explanation, which has hitherto been proposed.—*Rev.*

### Illustrations of Abdominal Pathology.

### Cases of Abscess of the Liver—in one Instance associated with Hydatids—communicating with the Lungs.

A middle-aged man was seized, in the Summer of 1827, with violent inflammation of the liver. Jaundice came on, and with it the usual symptoms of gall-stones. For several years afterwards, his health was every now and then interrupted by bilious attacks, especially when he was exposed to any mental vexation or excitement. In 1834, he suffered more than usual from these attacks; and, in 1835, he had a severe seizure of acute hepatitis. The inflammatory symptoms on this occasion were succeeded by those of suppuration. On awaking one morning, he was seized with a most distressing hiccup and sense of syncope; and immediately afterwards with a cough, followed by the expectoration, or rather the vomiting, of a large quantity of a greenish-coloured and offensive fluid. The right hypochondrium, which previously had been swollen and painful, immediately became easy and of its natural fulness. At first, it could not be well determined whether the discharged fluid came from the lungs or from the stomach; but the teasing cough and copious expectoration, which continued to harass the patient, naturally led to the suspicion that the lungs were at fault.

The patient felt himself much relieved for some time: but still the fever continued, and was accompanied with a most distressing sensation of burning in the throat and along the larynx. On auscultation, it was found that, immediately under the clavicles, the respiratory murmur was very dull; lower down, on the right side, a mucous râle was audible, and, still lower, the râle became cavernous, and the chest sounded unusually resonant. It was, therefore, suspected that one or more caverns existed in the lower lobe of the right lung, and that these probably communicated with an abscess of the liver. The patient rapidly sunk, and he died on the 5th of October.

On dissection, the liver was found much enlarged. On cutting through the right lobe, a large cartilaginous cyst, filled with a yellowish-brown, thick and fetid fluid, in which there were numerous hydatids, was exposed. Some of these entozoa were still entire, and of the size of a bean or hazel-nut; others were flattened and irregular, and looked as if they had formed parts of hydatids as large as a hen's egg, or even as an orange. On the upper part of the cyst there was a large aperture, which was found to communicate with a cavern in the lower lobe of the right lung. The parietes of the pulmonary cavern were soft, covered with flocculi, and contained a fluid similar to what was found in the hepatic cyst.—*Caspar's Wochenschrift, Mai,* 1836.

To illustrate the occasional discharge of hepatic abscesses by the way of the lungs, we shall briefly narrate the histories of two cases, recorded by Dr. Stokes in the Meath Hospital Reports.

A patient, who had just recovered from a very severe attack of gastro-bilious fever, lapsed into a state of hectic fever. As this was attended with a troublesome dry cough, the chest was repeatedly examined; but no unnatural sound could be discovered on either side, by percussion or even by auscultation. Suddenly he was seized with a feeling of suffocation, and he began to expectorate large quantities of purulent matter. Next day, the lower part of the left side of the chest was found to be quite dull on percussion, and no respiratory murmur, or râle of any sort, was to be heard there. The expectoration continued for some time; and then the auscultatory phenomena were observed to be very different from what they had been on the former examination. First, a mucous rattle was heard over the lowest part of the lung; this gradually extended higher and higher up; and, by degrees, the respiratory murmur and resonance of the voice became audible over all the extent of the side, where previously they had been quite extinct. As this patient recovered, we cannot be quite assured of the correctness of Dr. Stokes' diagnosis.

The other case affords an example of an abscess rather *on*, than *in*, the liver, discharging its contents upwards through the diaphragm into the lungs.

A young woman, long affected with amenorrhœa, was attacked first with hæmoptysis and cough, and subsequently with pain in the right hypochondrium, much increased by pressure, by deep inspiration, and by any motion. The sound on percussing the lower part of the right side of the chest was very dull, and the respiratory murmur there was feeble and indistinct. In the course of a fortnight, a diffused inflammatory swelling began to occupy the right hypochondrium. This was poulticed for several days, and then it was opened with a lancet. A large quantity of pus, mixed with blood, flowed out. The matter again accumulated, and it was necessary to make another opening for its discharge. The wound now assumed all the characters of an anthrax; and the patient's strength began rapidly to decline.

On introducing a probe, it was found to pass deep under the muscles in several directions; but whether it entered the cavity of the pleura, or of the peritoneum, was quite uncertain. The respiration in the infra-mammary region was cavernous, and accompanied with a sound like the ticking of a watch. When the patient coughed, or breathed very deeply, a loud gurgling noise was heard. On dissection, the fistula from the outward wound was found to pass between the eighth and ninth ribs to the convex surface of the liver, on which, but external to its peritoneal covering, rested the base of the abscess. This abscess communicated upwards through an ulcerated portion of the diaphragm, with a purulent cavity in the lower lobe of the right lung.

Besides the above cases, we shall briefly notice one detailed in the clinical report of Dr. Gorgone of Palermo (the receipt of which we acknowledged with thanks in our last Number.)

A woman, 36 years of age, presented on the examination of the right hypochondriac region a swelling, which was firm, painful, smooth and uniform, circumscribed, and somewhat elevated towards its centre. By some of the surgeons, it was supposed to arise from a solid enlargement, or from an abscess of the liver, while others regarded it as an encysted dropsical sac.

Dr. Gorgone, finding that an indistinct fluctuation was perceptible, pronounced the case to be one of abscess, situated on the concave surface of the liver. He punctured it with a trocar, and gave issue to a quantity of offensive pus, in which there were several small hydatids floating about. The opening was afterwards enlarged with a bistoury: the discharge flowed copiously for several days, and during that time, upwards of 300 hydatids escaped from the wound. The patient gradually recovered, and was pronounced to be cured in about two months after the date of the operation.

CASES OF ABSCESS IN THE ABDOMINAL PARIETES. By Dr. GORGONE, of Palermo.

CASE 1.—*Abscess in the Hypogastric Region.*

A young female had been for a considerable length of time annoyed with a hard, unyielding, somewhat uneven tumor, situated in the hypogastric region, immediately over the bladder. Dr. G. and another surgeon regarded it as proceeding from an enlargement of the uterus. In course of time, however, an obscure fluctuation was felt in different points of the swelling; and, as the patient's health was now affected with symptoms of hectic disturbance, it was determined to make a deep incision into the most prominent part ("interassando," adds our author, "tutta la spessezza della linea bianca ed il peritoneo:" Are we to understand from this that he divided the peritoneum?), to ascertain the true nature of the swelling. Upwards of three pints of purulent matter were discharged. A troublesome fistula remained for a great length of time, in consequence of the matter having burrowed down at the side of the bladder. The use, however, of tent bougies, and the occasional application of the nitrate of silver,* gradually effected an entire and permanent cure in this case.

Case 2.—*Abscess of the Abdominal Parietes.*

A lady, 32 years of age, had for several years a swelling, of the size of a hen's egg, situated in the centre of the hypogastric region. It was hard, indolent, and occasioned but little uneasiness to the patient. Her medical attendant had pronounced it to be connected with an enlargement of the uterus.

After a smart attack of fever, the swelling became larger and more uneasy; and it now seemed to cause a difficulty in passing the water, and a sense of weight about the anus, and of torpor of the hips and thighs. A consultation of physicians was held, and it was given as their joint opinion that the swelling was *uterine*. By the use of antiphlogistic means, it was brought back to an indolent, inactive state; but soon afterwards the feverishness began to return in daily paroxysms. At first, this was believed to be an accidental attack of genuine intermittent, and quinine was administered. It was soon, however, discovered that the fever had much more of the characters of a hectic† than of an ague. The abdominal swelling gradually became softer to the touch, and an obscure fluctuation was perceived on pressure at several points, the rest of it being still hard and unyielding.

Two surgeons, who now examined the case, pronounced it to be one of encysted dropsy. One or two fresh attacks of inflammation in the tumor added greatly to the sufferings of the patient, and her strength was daily becoming less and less by the recurrence of the hectic fever. At this period Dr. Gorgone visited the patient for the first time. He found a large prominent swelling in the hypogastric region, reaching up as high as the umbilicus, and extending rather more into the left than into the right iliac fossa. It was prominent, smooth and uniform to the touch, soft, and presenting a sense of obscure fluctuation. The uterus was found, on examination per vaginam, to be sound. The opinion which Dr. G. gave of the case was, that there was a collection of pus; but whether the abscess was idiopathic and external to the peritoneum, or whether it was internal, and connected with any disease of the uterus, he could not decide. He proposed, however, to make an incision into the swelling for the purpose of discharging its contents. This advice was opposed by some of the other surgeons, who had been called into consultation. They still regarded it as, probably, an encysted dropsical swelling, and they recommended frictions with mercurial ointment, and the internal use of diuretics, in the hope of dispersing it. The swelling gradually increased in size, and the left limb had now become œdematous and almost paralytic. After much delay, Dr. G. was permitted to cut into the swelling. The integuments being divided, he "cut through the linea alba to the extent of an inch, and the abdominal parietes along with the peritoneum." Nearly six pints of unhealthy-

---

* In the management of such cases, when we wish to use the nitrate of silver, it will be found to be a most convenient plan, to melt a small portion of the metallic salt in a spoon, and immerse the blunt end of a probe in the liquid caustic. It dries almost immediately, and we thus procure a coating of nitrate of silver on the point of the probe.

† We have seen this very serious mistake committed oftener than once in practice. We well remember seeing, several years ago, a case of *disguised* phthisis—disguised by the absence of cough and expectoration—in the last stage, that of purulent excavation, where the early attacks of afternoon hectic fever were mistaken for imperfect paroxysms of ague, and treated with quinine—we need not add, unsuccessfully. The mistake was the more excusable, as the patient had recently come from a marshy district.—Rev.

looking offensive pus were discharged. For several days successively, upwards of a pint of matter flowed out from the wound. As the sac was so very large, it was necessary to employ graduated pressure over it with great nicety and care, and to make one or two counter-openings at those parts, where the pus was apt to lodge. Setons were introduced between the openings, for the purpose of exciting action in the walls of this large cyst, and injections of a solution of alum were used daily. The progress of the case was highly satisfactory. By the use of mercurial and of iodine frictions on the abdomen, of quinine and other tonics taken internally, and with the advantage of a pure country air, the patient was ultimately restored to perfect health.

---

### Memorable Case of Spontaneous Perforation of the Bowels—Sero-purulent Effusion in the Abdomen—Life prolonged by repeated Paracentesis—Ramollissement of the Urinary Bladder.

The following case appears to us remarkable, for the prolongation of life under the most unfavourable circumstances. It is narrated at great length by M. Piorry himself, under whose care the patient was, in the Bulletin Clinique for June 1836.

A young man, 20 years of age, had been ailing from diarrhœa, vomiting, and other symptoms of general malaise, for several weeks before his admission into the Hôtel Dieu. There had been also a gradual tumefaction of the abdomen, coming on during the preceding five or six days. When admitted, the patient was in a weak typhoid state; the distention of the abdomen was very considerable, and this distention had mounted so high, that the heart seemed to have been pushed up much nearer to the clavicle than in health. The sense of fluctuation was indistinct, and the sounds elicited by the percussion of the abdomen varied in different parts—in some places being quite dull, in others resonant.

The diagnosis, which M. Piorry formed, was that the case was one of "enterite typhohemique et hydro-peritonite." He recommended that the abdomen should be tapped. On withdrawing the trocar, only a few drops of serosity flowed out from the canula. By inserting, however, an elastic gum sound through the canula into the abdomen, and moving its point freely about in different directions, three pints of a sero-purulent fluid, having an odour partly fæculent and partly of sulphuretted hydrogen, escaped. The operation lasted upwards of an hour.

M. Piorry was now more satisfied of the correctness of his diagnosis, that a perforation of some portion of the bowels had taken place at the period when the abdomen had begun to swell; and he conjectured that this perforation was in the small intestine, near the cæcum. On the day following, the 19th April, another puncture was made, and four pints of a fluid, quite similar to that already evacuated, escaped by the canula. The operation was again repeated on the 21st, and also on the 22d: "le liquide etait encore de la même nature, et presentait la même odeur; seulement il etait plus épais et plus jaune." For several days subsequently to the last operation, the state of the patient was in all respects better; but a diarrhœa then set in, and the tumefaction began to increase. On the 5th of May, the paracentesis was again performed; and, this time, some gas escaped along with a pint and a half of a yellowish, opaque, and fetid serosity. The patient lingered on to the 14th.

On dissection, the abdomen was found to contain between three and four pints of a highly offensive, yellowish-green serum. Almost the whole surface of the abdominal peritoneum was thickened and lined with a dense layer of pseudo-membranous deposit, soft, and of a yellowish colour on the surface; but which had not, in any part, contracted adhesion to the intestines, even at those points where the punctures had been made. The intestines were collected into an agglutinated mass in the ileo-cœcal region. On examining the mucous surface of the large gut, the glandulæ Brunneri were found inflamed and ulcerated. At one point, just at the commencement of the colon, there was a large patch of these tumefied, livid, and ulcerated follicles, and at this point there was a perforation, large enough to permit the point of the little finger to pass fairly through all the coats of the intestine. Another perforation, but of much smaller size, was found in the ileum before it joins the caput coli; and, on slitting open the gut, the mucous surface in different parts was found corroded by numerous superficial ulcerations.

The only other morbid lesion, which we shall notice, was the remarkable degree of ramollissement which the urinary bladder had undergone. So soft had its parietes become, that the mere weight of a probe was sufficient to penetrate them.

*Observations.* M. Andral has narrated, in his Clinique Medicale, a case, where the patient survived for a length of time, after a spontaneous perforation of the intestine and of the abdominal parietes. Dr. Stokes of Dublin has lately communicated the histories of some cases, to shew the good effects of large doses of opium in arresting the development of abdominal inflammation under such alarming circumstances; and M. Chomel has adopted the same practice with advantage.

That the life of our patient was greatly prolonged by the repeated paracentesis cannot, we should think, be doubted. We are not aware that any such case has been put previously on record.—*Bulletin Clinique.*

---

### On the Enlargements of the Spleen, after Agues and other Fevers.

Before we discuss the question of the probable cause of those enlargements of the spleen, which are so often the consequence of intermittent fevers, it may be useful to glance at some of the most curious and best ascertained facts connected with the healthy anatomy of this viscus. To Professors Felici and Panizza of Pavia, the profession is indebted for an admirable monograph on this subject; wherein they have most indisputably demonstrated the analogy of the texture of the spleen, with that of erectile tissues in general, and especially of the corpora cavernosa of the penis and clitoris. The character of this texture may be exhibited, either by repeatedly washing a portion of the organ with pure water, until it is completely freed from all the blood which it contains; or by blowing air along the splenic vein, so as to distend the cells. The latter of these methods will be found to succeed better when the spleen is enlarged, than when it is, from any cause, contracted in its dimensions. In our brief examination of the anatomy, we shall commence from the exterior of the viscus and proceed inwardly. The outer envelope of the spleen is its serous or peritoneal coat; this adheres firmly to the subjacent fibrous tissue, the tunica propria, or proper investing capsule, which binds or confines the whole organ together in one mass, and from whose inner surface are sent off numerous prolongations, some of which dip between the different portions, or lobules, forming the septa, or partitions between these, and others, by joining and rejoining with each other, give rise to the cellular, or reticular texture, which pervades the whole viscus. If the spleen be hypertrophied, the cells may be seen with the naked eye; but, in its natural state, the use of a magnifying glass is necessary to discover them.

The tunica propria, and all its prolongations are evidently very contractile; for if we cut a fresh spleen into slices, the contraction of the septa causes all the contained fluid to ooze out, and the divided surface then exhibits a granulated appearance, similar to what we observe in a portion of lung, which is in the second stage of peripneumonia.

The arterial distribution of the spleen deserves particular notice. No sooner do the branches of the splenic artery enter the substance of the viscus, than they become so minutely subdivided, that they can no longer be traced. Assolant has proved, by a multitude of experiments, that, if one of the branches of the splenic artery be tied, the portion of the organ, on which the branch is distributed, is inevitably killed; and he infers therefore that there is but a very imperfect anastomosis between the bloodvessels of the spleen. This is confirmed by the fact, that if air is blown into one of the arterial branches, it does not pass freely, if at all, into the others. The anatomy of the veins of the spleen is better understood. They commence by extremely minute twigs, which are continuous with those, of which the cellules are formed, and of which they appear to be the mere prolongations; several of these twigs uniting give rise to larger branches, whose parietes are perforated with numerous apertures, large enough to admit a probe, and which communicate directly with the cellules. These apertures become smaller and less frequent, as the branches approach the large divisions of the splenic vein, which are usually three or four in number, and which meet at the concave edge of the spleen, into one large trunk: this proceeds on to meet the vena portæ. The lymphatic vessels of the spleen are neither numerous, nor very conspicuous. They are discovered much more easily on its surface, than within its texture. The nervous supplies are derived partly from the cœliac plexus, and partly from the par vagum.

Without entering upon the labyrinth of theories, as to the uses and functions of the spleen, we may be permitted to say, that it is, at least, probable, from the analogy of its structure with that of the recognized erectile tissues, that there is some substance interposed between its vasa sanguifera, *inferentia, and efferentia:* unless we admit M. Andral's opinion that "la rate n'est qu'un reseau veineux, où la forme celluleuse tend à remplacer la forme vasculaire." Whether, however, the spleen is to be considered as a mere diverticulum to the blood of the abdominal viscera, during certain states, or operations of the system; or whether it has not at the same time more elaborate duties to perform, (as for example being accessary to the formation of bile, or to the production of blood, either directly, or by generating a peculiar sort of lymph, which is afterwards conveyed by the absorbents into the thoracic duct) remains still a problem of most difficult solution. After these short preliminary remarks, we now approach the question, as to the probable cause of the enlargements of the spleen, which, it is well known, are very frequently induced by intermittent fevers.

Some pathologists have attributed these lesions to a merely passive or atonic accumulation and congestion of blood in the veins or cells of the viscus; and they have altogether excluded the idea of any active increase of vascular action. The cause, according to them, is therefore purely mechanical; the normal contractility of the veins, or cells being supposed to be overcome by the excessive distention of their parietes.

But surely this mode of accounting for any change in the condition of a living organ is not very consistent with what we observe in other parts of the body. What viscus can be alternately overcharged with, and drained of blood, without its vital energies being affected, or without some reaction being induced? And again, when we consider the variety of morbid degenerations, to which the spleen is subject, such as, induration, ossification, softening, conversion into a black pultaceous mass, the formation of genuine purulent matter, and the adhesion of its surfaces to the surrounding organs, surely we shall be forced to believe that some other morbid process, besides that of mere congestion of blood, may affect its substance. Moreover, if the engorgements of the spleen which occur during the course of intermittent fevers, were owing altogether to an excessive accumulation of blood in the vena portæ, we might naturally expect, that some of the other viscera, whose veins terminate in the same trunk with those of the spleen, should be almost always similarly diseased at the same time.

Occasionally, indeed, the liver as well as the spleen is found enlarged; but this is by no means of constant, nor indeed of very frequent, occurrence. Morgagni was of opinion that the liver is oftener contracted than enlarged in such cases; and on the other hand it is worthy of notice that in the majority of the examples of diseased liver, recorded and described in Andral's Clinique Medicale, no notice is taken of any co-existent lesions of the spleen; and that, out of the 187 cases mentioned in Lieutaud's Anatomie Medicale, 23 of them only were complicated with splenic alterations.

The very obvious inferences from these facts are sanctioned and confirmed by the physiological consideration, that, as the veins of the spleen are so much larger and more numerous than its arteries, it is not easy to understand, how a stasis of the blood, or a passive congestion can take place in them, unless there be some obstruction in the vena portæ, or vena cava, to the centripetal blood. Such a complication may occur, and does, we know, exist in cases of diseased heart; in which there is a most distressing tendency in almost every organ to a morbid accumulation of venous blood. Hence the source of the apoplexies, dropsies, dyspnœas, engorged livers, hæmorrhoids, &c. which are so frequently observed in the progress of cardiac cases.

But, although these reasons may be considered satisfactory to shew that enlargements of the spleen do not depend upon a mere passive congestion of the venous blood in its texture, the question, what is the cause, or rationale of the afflux of the blood from the surface of the body, to the internal parts, and more especially to the spleen in agues? now presents itself to our consideration. Cullen's hypothesis is well known to most medical readers: the primary spasm of the peripheral vessels, the contraction of their diameters, and the consequent expulsion of part of their contents, &c. from the surface to the deeper organs are its leading features. The first movement in the morbid action is, according to Cullen, in the vessels of the skin, and the internal congestions are the necessary and almost mechanical effects of the blood being driven from the skin inwards. Dr. Fallot, the author of the present paper, assumes it to be more consistent with the operations of living bodies, that wherever a sudden alteration in the quantity of blood in any part or organ takes place, we may be satisfied that some change in the innervation of that part or organ has already preceded it; and that it is not the result of a merely mechanical afflux from one point to another. "*Ubi stimulus ibi affluxus*," is a physiological maxim, to which there is no exception; and the converse of the proposition is equally true, that where the excitement is defective, there is the afflux of blood diminished.

If we observe what takes place during the re-action of an ague, we shall find that the feeling, or consciousness, to the patient at least, of the hot stage very distinctly precedes the full throbbing pulse, and warmth of the skin, which are the indications to others of its presence. Shall we then make an exception of the spleen, and consider it as nothing but an inert sponge, at one time soaked with blood, and at another time empty, according as other organs have an excess, or deficiency of the circulating fluid? And when all other erectile tissues are uniformly subject to the law that we have already announced, viz. that of being engorged only when they are stimulated, and of becoming flaccid, when the stimulation is diminished or withdrawn, why shall we apply other laws to the spleen, and suppose that it is excepted from the vital actions of similar parts? We infer, therefore, that if the spleen becomes congested and enlarged during an intermittent fever, this congestion and enlargement are the effects of an abnormal afflux of blood, occasioned by the pre-existence of an abnormal stimulation in the part. Observe the changes induced in a splenic tumor in the phases of an ague. Soft, and but little painful during the intermission, it becomes distended, hard, and sensitive during the cold stage; but it is not, until the vascular re-action of the hot stage is fairly established, "quand les mouvemens d'expansion sont en pleine activité," that it attains its maximum of intumescence, resistance, and tenderness. When the irritation is slight or temporary, the vascular excitement subsides, and is gradually dissipated entirely, the swelling being no longer appreciable. When it is more severe, or is persistant for a considerable time, the blood is forced into the areolæ, or minute cellules, which then become so gorged and over-distended, that their parietes take on an inflammatory action, and this state is succeeded by one of induration, or by the deposition of purulent matter. Under these circumstances the spleen, from its augmented bulk, is felt projecting below the edges of the false ribs on the left side.

Supposing now, that the question which we have been considering, viz., whether the enlargements of the spleen after agues are generally the result of an active, or of a passive congestion of blood in its texture, to be decided according to the views explained above, another of almost equal difficulty immediately presents itself. This active congestion—is it idiopathic and independent of the affection of other viscera; or is it merely sympathetic, and consecutive on some irritation existing elsewhere?

On the one hand, it may be alleged that the diseases of the spleen (with the exception, perhaps, of the enlargements now under discussion), are very rarely isolated, but are usually coexistent with lesions of other organs; and on the other hand, we must recollect that our ignorance of the healthy functions of this viscus precludes us from detecting its morbid deviations, unless these are accompanied by obvious and visible changes of structure. The subject, therefore, of splenic disease must be one full of uncertainty and difficulty. M. Broussais, in treating of the diseases of the liver, has rendered it probable that this important viscus is very seldom primarily or idiopathically affected, unless from an external injury. What is probable of the liver, is much more so of its neighbour, the spleen. The frequent co-existence of splenic disease with diseases of the heart deserves the attention of morbid anatomists, and has not received that minute investigation, which the importance of the subject requires. Dr. Kreisig, of Dresden, who has written a good treatise on Die Krankheiten des Harzes, explains the connexion of splenic with cardiac disease, by reference to the obstruction offered to the free return of the blood in the venæ cavæ, and to the consequent congestion, which all the abdominal viscera must experience under such circumstances. This explanation, though satisfactory to a certain extent, cannot well apply to every case of diseased spleen. According to Kreisig himself, alterations of the spleen are frequently co-existent with diseases of the left, as well as of the right, side of the heart; and Testa, and other Italian physicians allege that many cardiac affections are secondary to, and are apparently induced by, morbid states of the spleen; although this viscus may not have become increased in bulk, and cannot therefore be supposed to have excited any mechanically injurious effect on the free circulation of the blood through the heart and large vessels.

Baron Alibert has suggested in his Nosographie Naturelle, that this sympathy between the spleen and heart may be accounted for, by their nervous connexion, through the medium of the par vagum, which he thinks " partage avec le grand sympathique la faculté de produire des sympathies." Without denying the importance of this connexion, we cannot concede to it the exclusive influence, assigned to it by the Baron: else how comes it, that the liver and stomach, which derive part of their nervous supplies from the same source, viz., the nervi vagi, do not as frequently sympathise with diseases of the heart, as the spleen is known to do? Dr. Fallot is, therefore, induced to believe that it is rather in consequence of it (the spleen), being an accessary or auxiliary instrument or agent of the circulation, and designed by nature to avert, and in some degree to compensate for the accidents that might ensue from any interruption of this vital function, (whether this arises from an excess, or from a defect of activity), that it is so intimately associated with the heart and lungs. This functional association, or sympathy, is not nearly so obvious and appreciable during health, as when either of these organs is involved in disease.

If the heart and lungs become, from any cause, oppressed with venous blood —let us remember that this state occurs during every cold stage of an ague—and in an obstinate case, how frequent must this oppression or congestion have taken place—the spleen must very speedily suffer, in consequence of the column of blood pressing back upon every part of the portal system: and where do you think, asks M. Fallot, " qu'elle doive retentir plutot que dans la rate, unie au cœur par des liens de synergie pour l'accomplissement de la meme fonction ?"—Moreover, the great size of the splenic artery, and consequently the large quantity of blood constantly transmitted through it, must of a necessity predispose the organ to be affected with various morbid states. It is a subject of great pathological interest, to ascertain, what is the relation, as to cause and effect, between enlargements of the spleen, and the very frequently simultaneous occurrence of alterations in the mass of the blood. It is certainly a fact, known to every experienced physician, that when the spleen has been diseased for any length of time, a dyscrasis of the circulating fluids is almost invariably induced. We cannot afford room, at the present time, to enter upon the discussion of this very curious theme: the mere suggestion of it must be sufficient.—*Bulletin Medical Belge.*

It may be useful to subjoin a few comments on some of the statements in M. Fallot's paper, and more particularly upon the suggestion made at its close. It is not quite easy to determine, whether the dyscrasis of the circulating fluids is owing to the morbid state of the spleen, or whether the converse of the proposition be true. However this may be, the constitutional symptoms usually associated with chronic enlargements of the spleen are these: general debility, paleness and a deficiency of red blood in the capillaries; the conjunctivæ are pale and bloodless, the scleroticæ are of a pearly blue colour, and the complexion is waxen, as in chlorosis.* If the disease has existed for some time, the extremities are apt to be cold, and the skin is pale, dry, shrivelled, and often furfuraceous. Adults do not usually suffer so much from splenic enlargement, as children do. In younger subjects, it is very apt to induce quickly an incurable marasmus ; whereas we not unfrequently observe that, with adults, it may exist for a length of time, without being attended with serious disturbance of the constitution. (Does not this difference seem to indicate that this viscus is probably more or less directly connected with the formation of the blood ?) Patients affected with vascular engorgements of the spleen are very prone to foul sloughing ulcers, from slight wounds or bruises; and all restorative processes are observed to be very tardily and imperfectly performed. The blood of such patients is always more or less unhealthy : sometimes it coagulates only imperfectly, and no serum is separated ; in other cases the cruor is black, soft, and does not assume a florid hue on the surface from the contact of the air. In short, not a few of the attributes or symptoms of genuine scorbutus are found in many cases of enlarged spleen. There is a tendency to hæmorrhage from slight wounds, or ulcers ; punctures and abrasions of the skin are apt to ulcerate ; the gums become gangrenous, the teeth fall out, and the alveoli become carious. Hæmoptysis and hæmatemesis are not unfrequent occurrences.

---

* The late Dr. Twining, of Calcutta, has very justly remarked that the constitutional disturbance which usually accompanies enlargements of the spleen, has many characters in common with chlorosis, scorbutus, and some forms of anæmia.—*Vid. Vol.* I. *of his Clinical Illustrations of the Diseases of Bengal.*

Besides the class of symptoms now alluded to, there are others which dedeserve to be noticed; such as the dyspnœa, (the blood seems to be imperfectly decarbonised,) and the tendency to panting and palpitation on any active exertion.

The functions of the stomach and bowels too are almost always disturbed; the food being not properly digested or assimilated, and the alvine evacuations irregular and unhealthy. There is moreover very generally a despondency and inactivity of all the mental powers.

In the advanced stage of the disease, dysentery or some form of dropsy usually supervenes.

The rapidity with which enlargements of the spleen sometimes occur after tropical fevers, and the enormous size which they may attain, are very striking.

It is very common not only to *feel*, but also to *see* it extending down on a level with the navel, and even lower. In extreme cases, we are told, the diseased spleen occupies more than half the abdominal cavity, extending to the right of the umbilicus and reaching into the left iliac region.

With respect to the treatment of splenic enlargement, it is unnecessary to state that the removal of the patient from the climate, where he has acacquired it, is in general by far the most efficacious remedy.

Should there be any feverishness of the system, moderate detraction of blood may be requisite, and the effects of this should be kept up by the exhibition of some active purges. When the enlargement is chronic and quite apyrexial, the use of purgatives, in union with bitters and with some form of steel, is by far the most successful treatment which can be adopted. Dr. Twining gives a formula for a compound, to which he gives the name of "spleen mixture," and which has been very successful in his practice. It is thus:—

℞. Pulv. jalapæ, pulv. rhei, pulv. calumbæ, pulv. zingiberis, potass. supertartratis, āā ʒj.

Ferri sulphatis, gr. x.

Tinct. sennæ, ʒiv.

Aquæ menth. sativæ, ℥x. Misce.

An ounce and a half of this may be given twice daily to an adult. Frictions over the affected side, and compression with a flannel roller, will be very generally useful at the same time.

Blisters are of very doubtful propriety. They are apt to be followed by troublesome ulcerations. The native practitioners of India have long been in the habit of employing acupuncturation in enlargements of the spleen.—Dr. Twining, whom we have already quoted, remarks in reference to this remedy: "I have repeatedly punctured indolent and indurated spleens with long needles—2, 3, or 4, being introduced exactly two inches in depth—and in some instances certainly with beneficial effects."

Lastly, with respect to that panacea of some English practitioners in all visceral diseases, mercury, it deserves to be impressed on the attention of every physician, that there is not perhaps a more dangerous or destructive practice, than that of exhibiting mercurials in most splenic enlargements. The constitution of the majority of patients, affected with this disease, seems to be unusually susceptible of the injurious effects of this mineral; and, therefore, as Dr. Abercrombie most justly remarks, "attempts to reduce enlargements of the spleen by mercury are generally followed by the worst consequences."—*Ed. Med. and Surg. Journ. Vol.* 22.—REV.

Hygiene Publique. Par A. J. B. Parent-Duchatelet. Deux Tomes, pp. 552 and 704. Bailliere, 1836.

[Continued from p. 410.]

M. Duchatelet next proceeds to enquire what influence the sewers have on the health of the workmen, employed in keeping them in repair. He first alludes to the effects which they produced upon himself. The usual symptoms, which he first experienced, were headache, and a certain degree of stupor, dryness of the throat, thirst, loss of appetite, and sometimes slight dyspnœa; but all these inconveniences speedily passed away, after he remained some time in the sewer. With respect to the general appearance and health of the regular workmen, he tells us that they are almost all thin, and rather meagre; their complexion is generally pale and earthy; the lines of the face are deeply marked;* the abdomen is usually much retracted;—yet, on the whole, the health of these people is "parfaite et fort rarement derangée." Their lives, too, do not seem to be at all shortened by their apparently most unhealthy occupation. Many of them suffer from lumbago and sciatica,—in consequence, probably, of the wet to which they are exposed, and also of the bent position, which they are so often obliged to maintain for a length of time. They are, he assures us, very rarely the subjects of cutaneous disease, or of any of those maladies which have been called "putrid!" But, from numerous observations and enquiries, M. Duchatelet is satisfied that all venereal complaints are greatly aggravated in the workmen, if they continue their occupations. Indeed, it has been very often remarked, that an impure atmosphere, of any description, is exceedingly unfavorable to the recovery of syphilitic patients: the disease generally becomes worse, and the only satisfactory method of effecting a cure is removal into the wholesome air of the country.

The same observation was made, forty years ago, by Parmentier and others, in their memoir on the health of the "vidangeurs," or those employed in cleaning the "fosses."

The late distinguished M. Hallé, who probably did more for the hygiene of Paris than any other single individual, confirms the truth of the statement, that syphilitic disease is always exceedingly obstinate and severe in these men.

But of all the dangers, to which the workmen are exposed, none is so alarming as that of asphyxia from the air becoming vitiated with azote, sulphuretted hydrogen, and other irrespirable gases. The symptoms, accompanying the attack of asphyxia, vary somewhat according to the nature of the gas, which has caused it. If it has been the ammoniacal sulphuretted hydrogen, the person is seized very suddenly; he becomes quite insensible in the course of a minute or so, and death is usually preceded by convulsions of the limbs. On the other hand, the attack is much more slow and gradual, when the asphyxia has been induced by azote: the breathing gradually becomes more and more embarrassed; vertigo and confusion come on; there are no convulsions; and the person is slowly suffocated. From what M. Duchatelet has observed, azote is the gas which is most frequently and most abundantly evolved in infected sewers.* The presence,

* These men seldom exhibited that bleared appearance of the eyes, so commonly observed in the regular scavengers, such as are employed in emptying the "fosses d'aisance." M. Duchatelet attributes this affection to the ammoniacal gas, evolved from putrefying fæcal matter. Now this gas does not exist, or only in minute proportion, in the sewers.

* The results of two analyses are these:—

In one, the proportion of azote was 94
of oxygen ............ 2
of carbonic acid, &c. gas 4

In the other, the proportion of azote was ........................ 89
of oxygen ............ 6
of carbonic acid, &c. gas 5

however, of the sulphuretted and carburetted hydrogen gases, must add greatly to the pernicious effects of the azote.

In 1826, one of the chief sewers in Paris, that of Amelot, became obstructed, in consequence, we believe, of its having been neglected for a length of time. Various attempts were made to clear it out; but none of these attempts had succeeded, and indeed most of the workmen, who had been employed, had suffered from more or less complete asphyxia, and some of them had lost their lives. The sewer was therefore left to itself; and became an insufferable nuisance to all the district, through which it passed. In course of time, it was completely stopped up; and then the contents flowed back upon the streets, and even into the courts of many houses in these streets. To add to the difficulties to be overcome, we ought to state that a new sewer had, a short time previously, been made to terminate in the obstructed one, and that all the blood, garbage, &c. from one of the chief "abattoirs" of the metropolis, as well as the water of many thousand sulphureous baths used at the Hôpital St. Louis, were discharged into the former. A commission, consisting of some of the most distinguished engineers and chemists, was appointed by the Prefect of the Seine to superintend the operations necessary for the "curage et l'assainissement" of these sewers. Our author was the secretary and reporter of this commission.

The workmen began their unpleasant operations at the mouth of the sewer, where it terminates in the Seine opposite to the "Ile Louviers." They were engaged for upwards of six months in this offensive and dangerous duty; but, in consequence of the admirable arrangements made, and the hygienic precautions which were taken, not a life was lost; although several of the workmen were, at different times, partially asphyxiated. By bringing them, however, at once into the open cool air, and exhibiting stimulants, most of them were quickly restored. In one case, after respiration was freely excited, a universal shivering, followed by convulsions of the limbs, came on: to these symptoms succeeded delirium, and then absolute mania; the patient roared and screamed aloud, tossed himself in all directions, and appeared to be quite insensible of those about him. After the operation of an emetic, his consciousness gradually returned, and in the course of a few days he had quite regained his former health.

Another man was brought to the surface insensible, pale, and motionless; the limbs being quite relaxed and the respiration suspended. By sprinkling cold water on the face, the breathing was restored; and then a few spoonfuls of an æthereal mixture brought back his consciousness and power of motion.

The directions, which M. Duchatelet gives for the treatment of persons asphyxiated by foul air, deserve to be shortly noticed. The person ought to be immediately brought into the open air, stripped of all his clothing, stretched out on the ground, with the head slightly thrown back; cold water should then be dashed smartly on the face and chest; vinegar or volatile alcali should be applied to, or injected into the nostrils, and the nostrils and throat should be tickled with a feather. Friction on the surface is also very useful.

If these means do not quickly succeed, then artificial respiration must be induced. M. Duchatelet has never seen any good, but sometimes serious mischief, from letting blood in asphyxia from breathing a foul air. He therefore, as a general rule, condemns the practice, until the patient has recovered from the immediate effects of the attack. This caution is the more necessary to be attended to, as medical men are far too apt to bleed indiscriminately in most cases of suspended animation—

---

M. Duchatelet has appended a table of twenty-one analyses of the air, taken from infected sewers. In every one of these, the proportion of azote was found to be greatly increased, and that of oxygen to be correspondingly diminished. We recommend physicians and chemists to examine the particulars of this table for themselves.

and we may add, of injuries of the head, and apoplexy. Whenever the act of breathing commences, the great difficulty is overcome; for, when once begun, it is easy to promote it by stimulants to the nostrils, by the feather introduced into the throat, and by tickling the soles of the feet. In the after-treatment of these cases, it is frequently necessary to apply leeches to the temples, or even to bleed from the arm, and to administer derivative medicines, to counteract the cerebral congestion.

We have already mentioned that some of the workmen suffered repeatedly from ophthalmia. This affection was transitory, if treated at once with astringent stimulating collyria. Sedative and emollient applications were of no benefit.

The attack of colic, which in some cases quite resembled in severity the worst form of colica pictonum, yielded generally to antispasmodic purgatives.

Before quitting this subject, we shall briefly allude to some of the most approved means to be employed by those, who venture into places where the air is much vitiated. The first thing to be considered is, whether it be possible to establish a current of atmospheric air through the place, by establishing a second opening in an opposite direction from that where the first is situated. If this can be done, the most ready and effectual mode of causing a free circulation of air is, to place a brazier of live coals between the two openings.

After ventilation, perhaps no means are more powerful in counteracting the deleterious agency of foul air, than chlorine and the chlorurets. The chlorine itself—obtained from a mixture of the oxide of manganese and of muriatic acid—is, for general purposes, too strong and suffocating to be employed while the workmen are in the sewer; but this objection is obviated by having recourse to its use, an hour or so before the men go to work. The chloruret of lime, however, on the whole is to be preferred. It yields a slow and very steady supply of chlorine; never too free, to incommode the breathing, and yet active enough to purify the surrounding air. Another advantage, which it possesses, is, that it may be used either in a dry or fluid form. One of the most convenient modes of using it by the sewermen and others, is to sprinkle a quantity of the dry powder on a heap of straw: it should be renewed four or five times in the course of the day. Another very good plan is to take a wide-mouthed bottle, and fill it with tow, on which the dry chloruret has been sprinkled. This bottle should be hung round the workmen's neck. Besides these precautions, it is useful to wash the face and hands repeatedly with the solution, and to throw a quantity of it on the offensive materials, which the men are working at.

Masks of various forms, and other similar contrivances have been suggested, with the view of enabling persons to pass through, and to remain in places, where the air is more or less vitiated.

During the plague, which raged at Marseilles in 1720, the physicians and other attendants on the sick were in the habit of using masks, which covered completely the head, and were provided with eye-holes, and with a long appendage, opposite the mouth and nostrils, and anointed on its inner surface with various balsamic substances.

The simple expedient of tying a piece of sponge, or folded linen well wetted with vinegar, over the mouth and nostrils, may often be used with much advantage. M. Fosse, who has paid much attention to the subject now under consideration, informs us that, with this simple means, he has been enabled to enter the most infected places, and to continue in them for 10 or 15 minutes at a time, without experiencing any inconvenience. The fluid, with which the sponge is wetted, should be different according to the nature of the deleterious emanations. Thus, when M. Fosse went into a chamber filled with the fumes of sulphur, he found that a solution of potash was most useful. On other occasions, when he was exposed to the abominable effluvia of sewers and privies, vinegar or a solution of the chloruret of lime was preferred. Lime water is probably the best preservative against carbonic acid fumes. Pure water will suffice to purify the air from matters, which are merely diffused mechanically through it, and to condense the vapours of metallic substances, as of mercury and arsenic.

To enable persons to remain for a greater length of time in an irrespirable atmosphere, it is necessary to have recourse to some means, by which a communication may be established either with the external air, or with a reservoir containing it. Such is the principle, it is well known, by which the men who go down in diving-bells are enabled to continue under the surface of the water for several hours at a time. On one occasion, at Bourdeaux, M. Duchatelet saw the workmen remain for nearly six hours continuously under water.

We may here allude to a very ingenious contrivance, proposed some years ago by an English workman of the name of Robert, to enable persons to enter into the rooms of a house on fire, and to remain in the suffocating atmosphere for a considerable length of time. The principle, on which this contrivance rests, is, that the air in rooms filled with smoke, is always much less vitiated near the floor than towards the ceiling. The apparatus consists of a mask, provided with eye-holes, which is put over the head, and secured with a tape round the neck, so as to prevent the admission of the surrounding air to the mouth and nostrils. A leather tube, four or five feet long, proceeds from the mouth-piece to which it is secured, and this hangs down to near the ancles; through it the person breathes. The open and inferior end of it is lined with flannel, or with thin pieces of sponge, which have been previously well wetted either with plain water, or with vinegar and water. The first experiments were performed in public at Manchester, in 1825. Robert and another workman, provided with their apparatus, went into a room in which a quantity of cotton had been burned, and which was quite full of smoky vapour: they remained in it for twenty minutes. On another occasion a quantity of moist straw, chips of wood, sulphur, and resin, was set fire to in the middle of a room, and the windows and door tightly closed, so that the room was quickly filled with the most suffocating smoke. Robert remained in it for upwards of half an hour. He was indeed considerably exhausted when he came out; but this exhaustion seemed to be owing rather to the high heat of the room, than to any embarrassment of the respiration. Numerous other experiments were performed with the apparatus both in this country and in France; and M. Duchatelet informs us that it has been (with certain modifications) so much approved of in Paris, that every body of firemen is provided with it. The chief objection to it—but one which may be very easily remedied—is, that the expired air is not got rid of, but enters the tube to be again inspired. If it was provided with a double, instead of a single, tube—the one for inspiration, and the other for expiration—the apparatus would be much more efficient. The reader will find a number of useful diagrams and descriptions of different instruments in M. Duchatelet's work.

Here we must stop in our analytic review. Happy we should have been, had the author been spared to meet the reward of praise and of profit, which his labours have so richly merited. But naturally feeble, and of a delicate constitution, the powers of his body were unequal to support the energies of his too active mind,

> "A fiery soul which, working out its way,

> Fretted the puny body to decay,

> And o'er-informed the tenement of clay."

His literary and scientific works will long remain a monument of his unwearied and most meritorious exertions.

